# Effect of modeling subject-specific cortical folds on brain injury risk prediction under blunt impact loading

**DOI:** 10.1007/s10237-026-02095-1

**Published:** 2026-06-19

**Authors:** Anu Tripathi, Alison Brooks, Traci Snedden, Peter Ferrazzano, Christian Franck, Rika Wright Carlsen

**Affiliations:** 1 Department of Engineering, Robert Morris University, Moon Township, PA USA; 2 Department of Orthopedics and Rehabilitation, University of Wisconsin–Madison, Madison, WI USA; 3 College of Nursing, University of Colorado Anschutz Medical Campus, Aurora, CO USA; 4 Waisman Center, University of Wisconsin–Madison, Madison, WI USA; 5 Department of Mechanical Engineering, University of Wisconsin–Madison, Madison, WI USA

**Keywords:** Mild traumatic brain injury, Finite element head models, Subject specific modeling, Cortical folds

## Abstract

Computational head models are essential tools for predicting the risk of mild traumatic brain injury (mTBI). However, computational models vary in the level of anatomical details, most notably the cortical folds. This study aims to determine the effect of modeling cortical folds on mTBI risk assessment. We compared gyrencephalic (with cortical folds) and lissencephalic (without cortical folds) finite element (FE) head models of 18 subjects aged 9–18 years, subjected to a rotational head acceleration of 10 krad/s$^2$ (10 ms duration) about each principal head axis. We analyzed the effect of cortical folds on different tissue-level mTBI injury metrics, including maximum principal strain (MPS95), maximum principal strain rate (MPSR95), and cumulative strain damage measure (CSDM15). The inclusion of cortical folds consistently yielded higher injury metrics across all individuals and rotational directions, with a bias (mean ± std. dev. relative to maximum lissencephalic values) of $21.7 \pm 9.1 \%$ in MPS95, $17.1\pm 7.6\%$ in MPSR95, and $14.4\pm 11.3\%$ in CSDM15. Differences in the spatial strain distribution were also found between the models, with the DICE similarity coefficient ranging between 0.07-0.43 and 0.42-0.70 for the peak MPS and CSDM15, respectively. Increases in peak injury metrics (up to ∼50%) were found for brain regions such as the corpus callosum, cerebellum, and brain stem. This study finds that the inclusion of cortical folds significantly alters the pattern of deformation in the brain and results in a prediction of higher mTBI risk.

**Supplementary Information:**

The online version contains supplementary material available at https://doi.org/10.1007/s10237-026-02095-1.

## Introduction

Mild traumatic brain injury (mTBI), including concussion, is a highly prevalent and heterogeneous condition that affects individuals differently (Langdon et al. 2023). Computational head models have been used to study the risk of mTBI for various activities across different populations (Liu et al. 2022; Filben et al. 2021; Takhounts et al. 2008; Nakarmi et al. 2025; Brooks et al. 2021; Zimmerman et al. 2021). These head models vary significantly in terms of the incorporated level of anatomical detail. One such anatomical detail that varies significantly between head models is the cortical folds, which consist of the gyri and sulci in the brain’s cerebrum. Some finite element (FE) studies include cortical folds in their head models (Reynier et al. 2022; Zimmerman et al. 2021; Miller et al. 2021; Filben et al. 2021; Li et al. 2021), whereas others use FE models without cortical folds (Cecchi et al. 2021; Brooks et al. 2021; Liu et al. 2022). The effect of incorporating cortical folds on mTBI risk assessment is not well understood. In this study, we aim to assess how the inclusion of cortical folds in subject-specific head models influences the predicted risk of mTBI from rotational head accelerations.

Previous studies have investigated the role of cortical folds on tissue-level mTBI injury metrics, such as strains (Song et al. 2015; Ho and Kleiven 2009; Sáez et al. 2020; Mazurkiewicz et al. 2021; Fagan et al. 2020) and stresses (Sáez et al. 2020; Fagan et al. 2020; Song et al. 2015) in the brain tissue. These computational (Song et al. 2015; Ho and Kleiven 2009; Sáez et al. 2020; Fagan et al. 2020) and experimental (Mazurkiewicz et al. 2021) studies compared the measured brain strains and stresses between gyrencephalic (with cortical folds) and lissencephalic (smooth cortical surface without folds) models, but resulted in conflicting findings. Some computational studies based on 3D (Ho and Kleiven 2009) and 2D sagittal models (Song et al. 2015) of the human head and 3D head models of multiple species (Sáez et al. 2020) reported higher strain and stress in the lissencephalic models. On the other hand, an experimental study on 2D coronal surrogates of the porcine brain (Mazurkiewicz et al. 2021) and a computational study on a 2D-axial human brain model (Fagan et al. 2020) found higher strains in the gyrencephalic models. These discrepancies could stem from differences in the impact loading conditions, tissue material properties, and the geometrical variations in the cortical folds modeled across the studies.

In addition to studying the effect of cortical folds on the brain deformation response at the whole head level, some computational studies have explored the role of geometrical variations in cortical folds on the deformation of the brain tissue at the mesoscale level (Cloots et al. 2008; Bakhtiarydavijani et al. 2021; Saboori and Sadegh 2012; He et al. 2022). These studies are based on 2D plane-strain models of a simplified gyri and sulci geometry. These studies also report conflicting trends, with most of the studies finding higher strains in gyrencephalic models (Bakhtiarydavijani et al. 2021; Cloots et al. 2008; He et al. 2022), but one study reporting higher strains in the lissencephalic model (Saboori and Sadegh 2012). The applied boundary condition used to approximate impact loading in these studies varied, with some studies applying uniform compression to the mesoscale model (He et al. 2022; Saboori and Sadegh 2012; Bakhtiarydavijani et al. 2021) and others applying a non-uniform shear acceleration field obtained from a full head simulation (Cloots et al. 2008). The studies also incorporate different tissue mechanical properties. Given these conflicting results, computational studies on mTBI continue to use both lissencephalic and gyrencephalic models to assess mTBI risk (Liu et al. 2022; Filben et al. 2021).

In this study, we investigate the role of cortical folds on predicted mTBI risk, while accounting for cortical fold variations across individuals. We develop subject-specific gyrencephalic and lissencephalic models of 18 subjects and compare their mTBI response under head rotations about the three principal anatomical axes. For each model, we assess common tissue-level mTBI metrics, such as maximum principal strain, maximum principal strain rate, and cumulative strain. By quantifying the differences in the mTBI metrics between the gyrencephalic and lissenphalic models, we can gain insight into the role of the cortical folds on the brain deformation response and the predicted risk of mTBI. Given the increased complexity and effort to create models that include cortical folds, it is important to evaluate the importance of including cortical folds in computational head models that are used to evaluate the risk of mTBI.

The paper is organized as follows. Section 2 describes the methods and workflows used to develop the subject-specific gyrencephalic and lissencephalic models from their medical images and provides details of the finite element simulations. Section 3 provides the results for the different mTBI metrics assessed in the computational study, and Section 4 discusses the implications of these results on our understanding and future steps.

## Materials and methods

In this section, we describe the development of gyrencephalic (with cortical folds) and lissencephalic (without cortical folds) finite element (FE) head models (Section 2.1), followed by details of the FE simulations (Section 2.2), injury metrics (Section 2.3), and data analyses methods (Section 2.4).

### Subject-specific head modeling

We developed FE head models of 18 individuals (8 males, 10 females; 9–18 years old) from their magnetic resonance imaging (MRI) scans. The MRIs were acquired at the Waisman Center at the University of Wisconsin-Madison. The acquisition was approved under the University of Wisconsin-Madison Institutional Review Board (IRB Protocol HSC2012-0069), and informed consent was obtained from each subject. Subject-specific gyrencephalic and lissencephalic models were generated for each subject. The number of subjects is listed by age and sex in Table 1.

**Table 1:** Number of subjects by age and sex

	9–13 Years	16–18 Years
Male (M)	5	3
Female (F)	6	4

#### Medical image segmentation

**Fig. 1:**
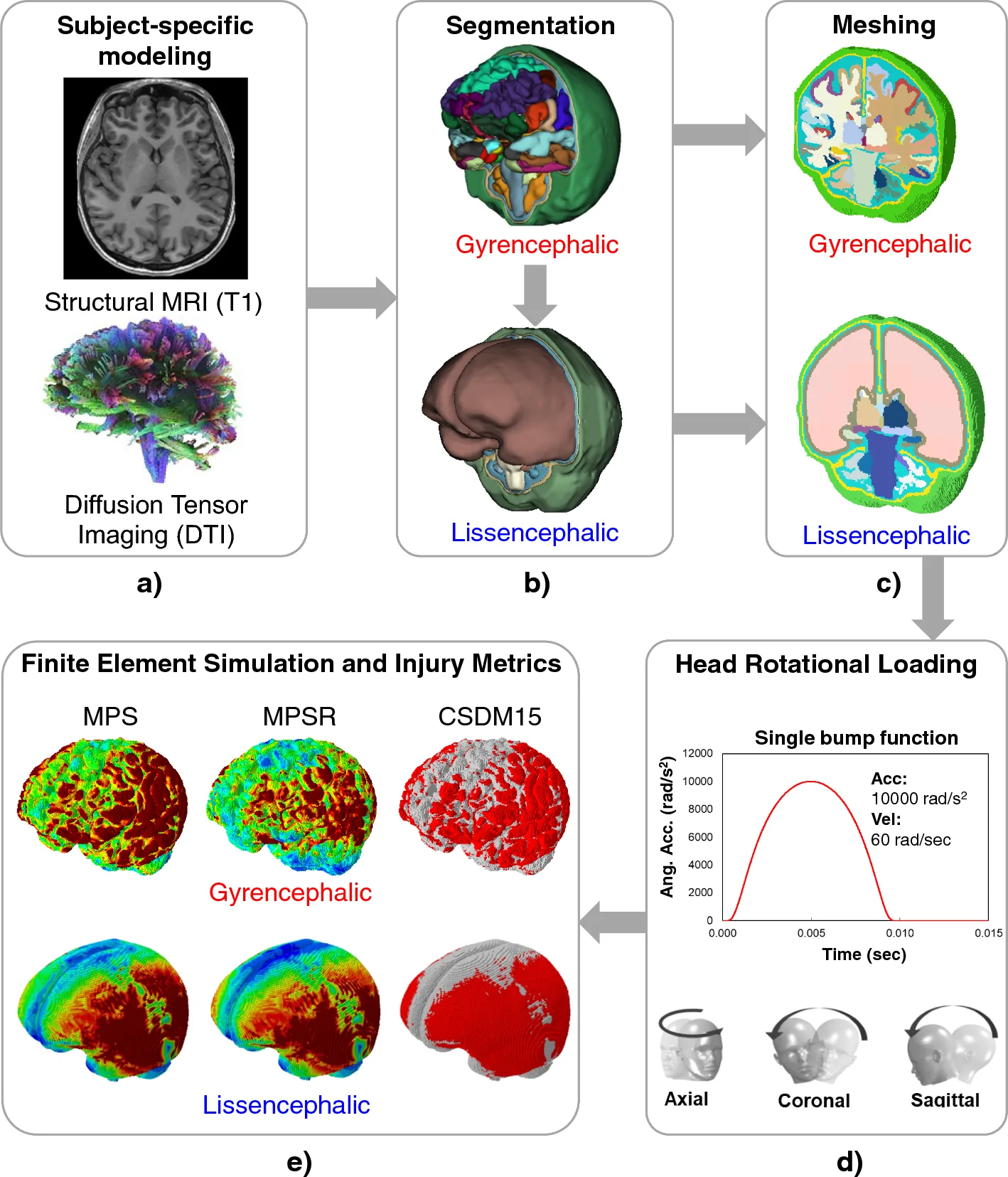
Workflow for studying the effect of modeling cortical folds on mTBI injury metrics: **a** Subject-specific magnetic resonance imaging (MRI) scans, **b** Medical image segmentation to create gyrencephalic and lissencephalic head models, **c** Voxel meshing, **d** Idealized head rotation acceleration profile applied about the three cardinal anatomical planes, and **e** Post-processing to obtain injury metrics

***Gyrencephalic Models*** The first step in generating a medical image-based FE model is medical image segmentation, which delineates the boundary of different parts of the model. The medical images of each individual consisted of structural MRI (T1-weighted) and diffusion tensor imaging (DTI) (Fig. 1a).

The T1-structural MRIs were first pre-processed to correct for Gibbs-ringing and bias-field artifacts using the open source software MRtrix (Tournier et al. 2019) and ANTS (Tustison et al. 2014), respectively. All MRI scans were registered rigidly to the MNI152 atlas using 3D slicer (http://www.slicer.org) (Fedorov et al. 2012). The segmentation of the brain tissue into the cortical gray matter, white matter, brain stem, and deep brain regions was obtained using the FreeSurfer software package (Fischl 2012). The resulting gray matter contained cortical folds (gyri and sulci) in 68 parcellations (Fig. 1b), providing a gyrencephalic head model segmentation. The skull segmentation was obtained by performing skull-stripping on the enhanced T1 MRI to obtain the skull mask using brain extraction tools (BET2) in the software FSL (Smith et al. 2004). The dura, falx, and tentorium segments were added manually after combining the FreeSurfer and FSL segmentations using 3D Slicer and were modeled to be 1 mm thick. The subarachnoid cerebrospinal fluid (CSF) was defined by filling in the space between the meninges and the brain tissue segmentations. A 1 mm layer of CSF was added around the meninges to ensure that the brain tissue was fully surrounded by CSF. This workflow provided the gyrencephalic model segmentations.

***Lissencephalic Models*** The lissencephalic models were generated by modifying the detailed gyrencephalic model segmentations. First, the heterogeneous subarachnoid CSF in the gyrencephalic segmentation was replaced by a uniform 1 mm thick layer of CSF lining the inner surface of the meninges. The cortical gray and white matter in the gyrencephalic segmentation were replaced by a uniform 4 mm thick gray matter layer inside the new uniform CSF layer segment. The remaining space between the gray matter and the deep brain structures was filled with white matter, to provide the lissencephalic model segmentations (Fig. 1b). It should be noted that the same material model was used for the gray matter and white matter (see Section 2.1.3), with the difference in properties originating only from the fiber dispersion parameter, which is obtained from DTI data and defined for each voxel of the brain tissue. Therefore, the material response is independent of whether a voxel is labeled as gray matter or white matter in the segmentation.

#### Finite element meshing

The FE mesh was generated directly from the segmentations by converting the segmented image voxels (1 mm × 1 mm × 1 mm) to hexahedral elements using a custom MATLAB script (Mathworks Inc.) (Fig. 1c). Each model consisted of $1.7-2.7 \times 10^6$ voxel hexahedral elements. A reduced integration scheme with hourglass control was implemented (element C3D8R) to prevent volumetric locking behavior given the nearly incompressible material behavior of the brain tissue.

A non-smoothed voxel mesh was chosen for this study because it allows fast mesh generation (∼15 minutes on 1 CPU) while retaining fine subject-specific details, such as the cortical folds. While the material interfaces are jagged in a non-smoothed voxel-based mesh, a recent study found that non-smoothed voxel, smoothed voxel, and conformal meshes (with smooth interfaces) provide nearly identical results when comparing strain based injury metrics, such as the 99th percentile strain or the volume fraction of brain tissue with strains over certain thresholds (Zhou et al. 2025). Furthermore, voxel meshes have been used in other FE head models with cortical folds (Ho 2008; Giudice et al. 2021; Miller et al. 2016).

#### Material modeling

The brain tissue was modeled as an anisotropic, visco-hyperelastic material with the axonal tract orientation and fiber dispersion obtained from DTI. The viscoelastic behavior of the brain tissue was captured using a first-order Prony series. The volumetric response of the CSF was modeled using the Mie Gruneisen equation of state, and the shear response was modeled as a Newtonian viscous flow. The meninges were modeled as a linear elastic solid, and the skull was modeled as a rigid solid. The material model definitions of the brain tissue, CSF, and the meninges were based on a previously published FE head model (Nakarmi et al. 2025; Wright et al. 2013). An evaluation of the brain mechanical response against experimental data for one of the subject-specific FE models used in this study is presented in Supplementary Sections S2 and S3.

### Finite element simulations

Three head acceleration events were simulated with each finite element head model, where a continuously differentiable bump function (Carlsen et al. 2021) rotational acceleration pulse was applied to the skull about each principal anatomical axis (axial, sagittal, and coronal). The peak acceleration was selected to be 10 krad/s$^2$ for a duration of 10 ms, resulting in a peak angular velocity of 60 rad/sec (Fig 1d). The loading magnitude was selected based on measured data in sports, such as mixed martial arts and American football, to represent an event with a high probability of concussion (Tiernan et al. 2020; Laksari et al. 2020). The simulations were conducted using an explicit time integration scheme in Abaqus FE software (Simulia, Dassault Systems). The simulations were performed on Abaqus/Explicit and each simulation took 10-14 hours using 64 cores.

### Injury metrics

To quantify the effect of including cortical folds on mTBI risk assessment, we compared the tissue-level injury metrics obtained from the gyrencephalic and lissencephalic model simulations for each subject. Previous studies have used measures of brain tissue strain and strain rate to evaluate mTBI risk (Gabler et al. 2016; Wu et al. 2021a; Margulies and Thibault 1992; Zhang et al. 2024). Therefore, we chose to assess the maximum principal logarithmic strain (MPS) and maximum principal logarithmic strain rate (MPSR) at each material element and time point from the FE simulations (Carlsen et al. 2021).

To assess volumetric damage, a cumulative strain damage measure (CSDM15) was also calculated, which is defined as the volume fraction of damaged elements to the total brain tissue volume (Takhounts et al. 2003). An element was flagged as damaged after the MPS at its location exceeded 0.15. The CSDM15 measure has been used in other studies (Carlsen et al. 2021; Takhounts et al. 2008; Ji et al. 2022), and the 15% strain threshold value is within the range of neuronal injury tolerance thresholds obtained from experimental studies (Hajiaghamemar et al. 2020; Bain and Meaney 2000; Bar-Kochba et al. 2016; Estrada et al. 2021; González-Cruz et al. 2024; Cullen et al. 2007). In addition to CSDM15, we also computed a cumulative strain rate damage measure (CSRDM50) based on elements exceeding an MPSR of 50 s$^{-1}$. This threshold value was chosen to be consistent with axonal strain rate tolerance levels defined in experimental studies (Hajiaghamemar and Margulies 2021; Rowson and Duma 2022).

Since the peak MPS at a given material point occurs at different time instances in the two models, we defined the time-invariant peak strain distribution for a given simulation as the peak MPS experienced by an element over the entire duration of the simulation (30 ms) (Upadhyay et al. 2024). We refer to this time-invariant peak MPS distribution as ‘the MPS distribution’ in the following sections, unless otherwise stated. This allows a comparison of peak strain experienced at the same anatomical locations between the two models, i.e., the similarity of the strain distribution. We also calculated the time-invariant peak MPSR distributions for a representative head rotation case.

To study the effect of cortical folds on the overall global response of the brain during a head acceleration event, we compared the highest strains and strain rates developed anywhere within the brain tissue at any time point during the simulation in the gyrencephalic and lissencephalic models for each given subject. The FE simulations were post-processed to provide the 95th percentile of the MPS (MPS95) and MPSR (MPSR95) instead of the 100th percentile, which is commonly avoided in complex biological FE models to avoid numerical artifacts.

### Data analysis

We use Sørensen DICE coefficient (DICE) to compare the similarity of the spatial strain distributions in the gyrencephalic and lissencephalic models. The DICE coefficient was obtained for the MPS and CSDM distributions, as defined below:

1 $$\begin{aligned} \text {DICE} = \frac{|G \cap L|}{|G|} \end{aligned}$$

where *G* and *L* are the element sets in the gyrencephalic and the lissencephalic models, |G∩L| is the intersection of the two sets, i.e., elements at corresponding spatial locations in both models that have the same MPS (or CSDM) values. We consider an element to have the same values in the two models if the absolute difference is less than 0.025. The DICE value is sensitive to this threshold, and we selected the value of 0.025 to be consistent with the contour plots showing MPS distribution in the results section (Section 3). A higher value of DICE indicates a better spatial agreement between the two models, with 1 indicating all elements have the same values and 0 being no elements have the same values. The DICE coefficient for MPS represents the similarity over the entire range of strain values, whereas the DICE coefficient for the CSDM represents the similarity in damaged elements in the two models.

We also calculated the means and standard deviations of the peak values of the injury metrics (MPS95, MPSR95, and CSDM15) from all subjects to compare the gyrencephalic and lissencephalic models. We performed this comparison across the whole brain and specific anatomical regions of interest (ROI), including the cerebrum, cerebellum, corpus callosum, brain stem, and deep brain regions comprising the thalamus, hippocampus, amygdala, putamen, pallidum, and hypothalamus. We performed a Bland-Altman test to obtain the bias between the gyrencephalic and lissencephalic models for peak MPS95, peak MPSR95, and CSDM15. The bias was obtained by subtracting the peak value of the lissencephalic model from that of the gyrencephalic model. A normalized bias was also obtained by normalizing the bias with respect to the maximum lissencephalic result. To evaluate statistical significance, we used a paired t-test, where p<0.005 is considered significant.

## Results

We developed subject-specific gyrencephalic and lissencephalic FE head models of 18 individuals from their MRI scans. We applied a concussive loading condition to each model through rigid body rotation of the skull (Fig 1d). In this section, we describe the tissue-level injury metrics (Section 2.3) obtained from these simulations to understand the effect of modeling cortical folds.

### Effect of modeling cortical folds on injury metrics: A representative case

**Fig. 2:**
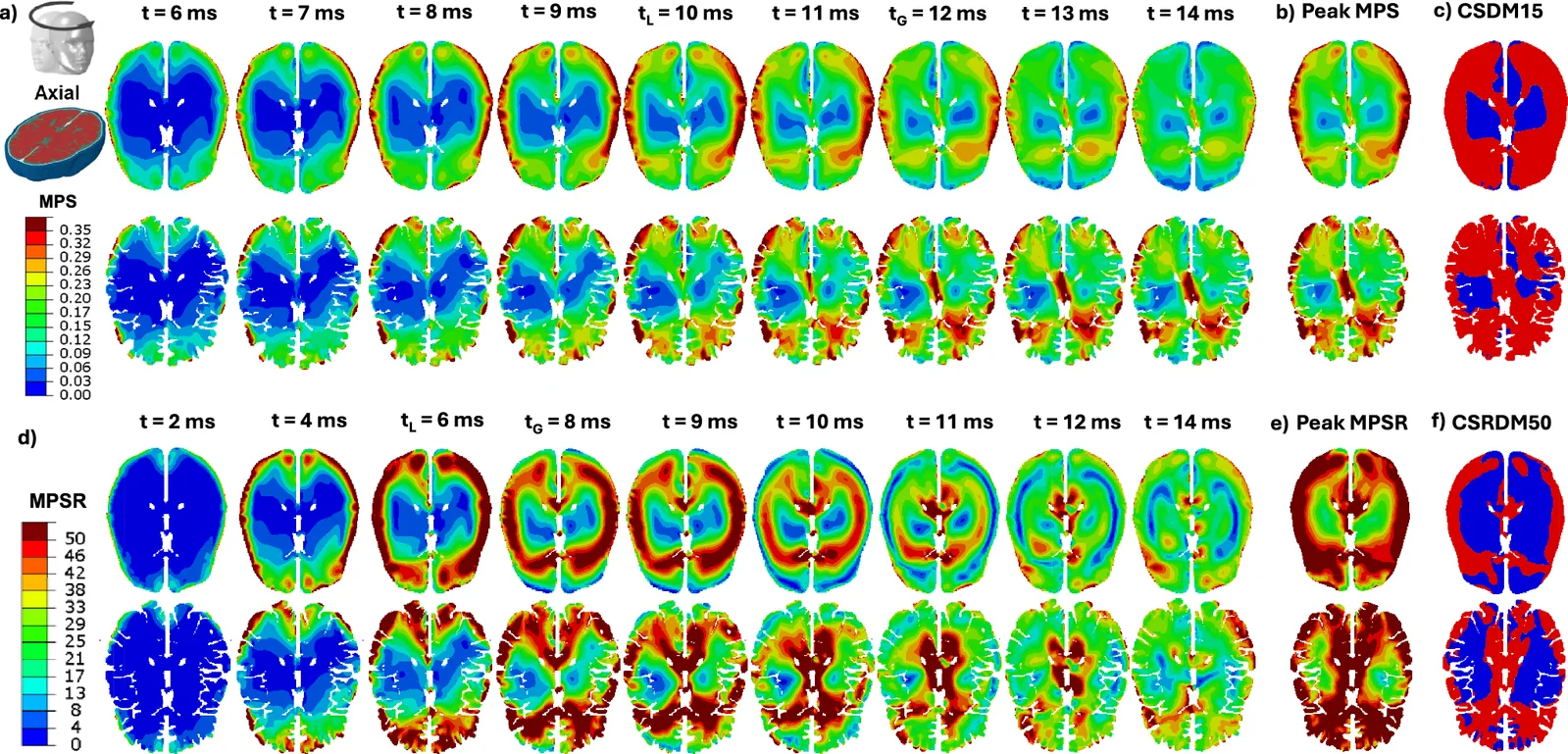
**a** Maximum principal strain (MPS) contour plots of a representative subject at different time points from the lissencephalic and gyrencephalic models ($t_L$ and $t_G$ are the time of highest MPS95 in the lissencephalic and gyrencephalic models, respectively). **b** The peak MPS experienced by the elements over the entire duration of the head acceleration event. **c** Damaged elements (red) where the MPS exceeded 0.15. **d** Maximum principal strain rate (MPSR) contour plots of the subject at different time points ($t_L$ and $t_G$ are the time of highest MPSR95 in the lissencephalic and gyrencephalic models, respectively). **e** The peak MPSR experienced by the elements over the entire duration of the head acceleration event. **f** Damaged elements (red) where the MPSR exceeded 50 s$^{-1}$. These plots highlight the spatial variations arising due to the cortical folds

This section analyzes the spatial evolution of the strain and strain rates during axial head rotation of a representative subject (18F) to identify the key differences in trends between the gyrencephalic and lissencephalic models (Figure 2). The spatial distributions are shown across an axial slice passing through the anterior and posterior horns of the lateral ventricles, parallel to the horizontal plane for the two models.

#### Brain tissue strain

We found some similarities in the propagation of the brain strain waves in the two models. The MPS distributions at different time points show that the strain begins to increase at the outer surface of the brain (cortical surface), and then the strain wave travels to the center of the brain in both the gyrencephalic and lissencephalic models (Figure 2a). We see high strain concentrations in the outer cerebral cortex in both models, although to different degrees (Figure 2a). Since a head acceleration event is modeled by providing the skull with a rigid body acceleration, the strain wave originates at the skull and travels inwards through the meninges and CSF towards the center of the brain. While the spherical convergence of the strain wave tends to increase the strain magnitude as the wave travels inwards, the high viscosity of the brain tissue causes dissipation of the strain energy, resulting in lower strain magnitudes, as the wave travels inwards. As a result, higher strains are generally found on the cortical surface (Figure 2a–f) (Massouros et al. 2014; Chen and Ostoja-Starzewski 2010).

We observed differences between the two models in terms of the location of the high strain regions on the cortical surface. The lissencephalic model experiences higher strain on the lateral surface of the brain, whereas the high strain regions in the gyrencephalic model are non-uniform.

In the gyrencephalic model, the space between sulci is filled with CSF, which is significantly softer than the brain tissue material (Wright et al. 2013). Therefore, modeling cortical folds directly impacts the effective stiffness of the skull-brain interface, which plays a significant role in the strains experienced by the brain tissue. The thickness of the CSF layer varies across gyri, contributing to the heterogeneity of the high-strain regions on the cortical surface. Also, the size and orientation of different sulci vary, resulting in different moments of inertia about the rotation axis, which can also affect the strain distributions. As the strain wave travels inwards, the geometric discontinuity from sulci (high curvature voids) causes strain concentrations at the deepest part of the sulci (fundus).

We observed further differences between the models as the strain wave reaches the tissue around the ventricles. The strain localization in the gyrencephalic model near the ventricles is significantly higher than in the lissencephalic model, especially between the anterior horns of the lateral ventricles and the Sylvian sulcus. While both models experience strain localization due to the high-curvature of the anterior horns of the lateral ventricles, the strain in the gyrencephalic model is further magnified in this region due to the close proximity of sulci, which lowers the effective modulus in the region. This region acts similarly to a single-edge notch specimen in the lissencephalic model and a double-edge notch specimen in the gyrencephalic model, yielding larger stress and strain concentrations in the gyrencephalic model. Given the larger strain in this region in the gyrencephalic model, the peak MPS95 was higher in the gyrencephalic model (peak MPS95=0.345) than the lissencephalic model (peak MPS95=0.288).

Overall, the lissencephalic model experienced the highest strains near the outer cortical surface of the brain, whereas the gyrencephalic model experienced the highest strains in the deeper regions of the brain near the ventricles, although the strains were also large at the outer cortical surface (Figure 2c). The difference in the spatial MPS distribution is reflected in the low DICE similarity coefficient of 0.22 between the models. The differences in the location of the largest strains also caused the time to peak MPS95 to be lower in the lissencephalic model ($t_L=10$ ms) than in the gyrencephalic model ($t_G=12$ ms). The CSDM15, on the other hand, which represents those regions of the brain exceeding 15% strain, had greater similarity between the two models (Figure 2d) with a DICE similarity coefficient of 0.67. It should be noted that since the CSF and brain tissue have the same density and the intracranial volume is identical between the gyrencephalic and lissencephalic models, the mass of the head is not changed. Therefore, the change in dynamics can be attributed to the changes in the stiffness and dissipation.

#### Brain tissue strain rate

Similar trends to MPS are found for the MPSR distribution when comparing the gyrencephalic and the lissencephalic models (Figure 2d–f). The MPSR distributions clearly show the strain rate wave propagating from the outer cortical surface towards the center of the brain, while dissipating and lowering in magnitude as it travels inward (Figure 2d). The lissencephalic model has higher MPSR and CSRDM50 on the outer cortical surface than compared to the gyrencephalic model (Figure 2e, f). The highest MPSR and CSRDM50 in the gyrencephalic model are near the center of the brain around the ventricles, due to the strain concentrations near the sulci and the ventricles, as explained in Section 3.1.1.

Similarly, we observe lower peak MPSR95 and time to peak MPSR95 in the lissencephalic model (peak MPSR95= 56.5 s$^{-1}$, $t_L=6$ ms) than in the gyrencephalic model (peak MPSR95= 67.9 s$^{-1}$, $t_G=7$ ms). Since the strain and strain rate differences follow similar trends, we only analyze the strain distribution differences across multiple subjects and loading directions in the following section.

### Inter-subject variability in injury risk due to modeling cortical folds

We analyze the injury metrics from the gyrencephalic and lissencephalic models for multiple subjects under different loading directions to evaluate the extent to which cortical folds influences mTBI risk assessments.

#### Effect of cortical folds on spatial strain distribution

**Fig. 3:**
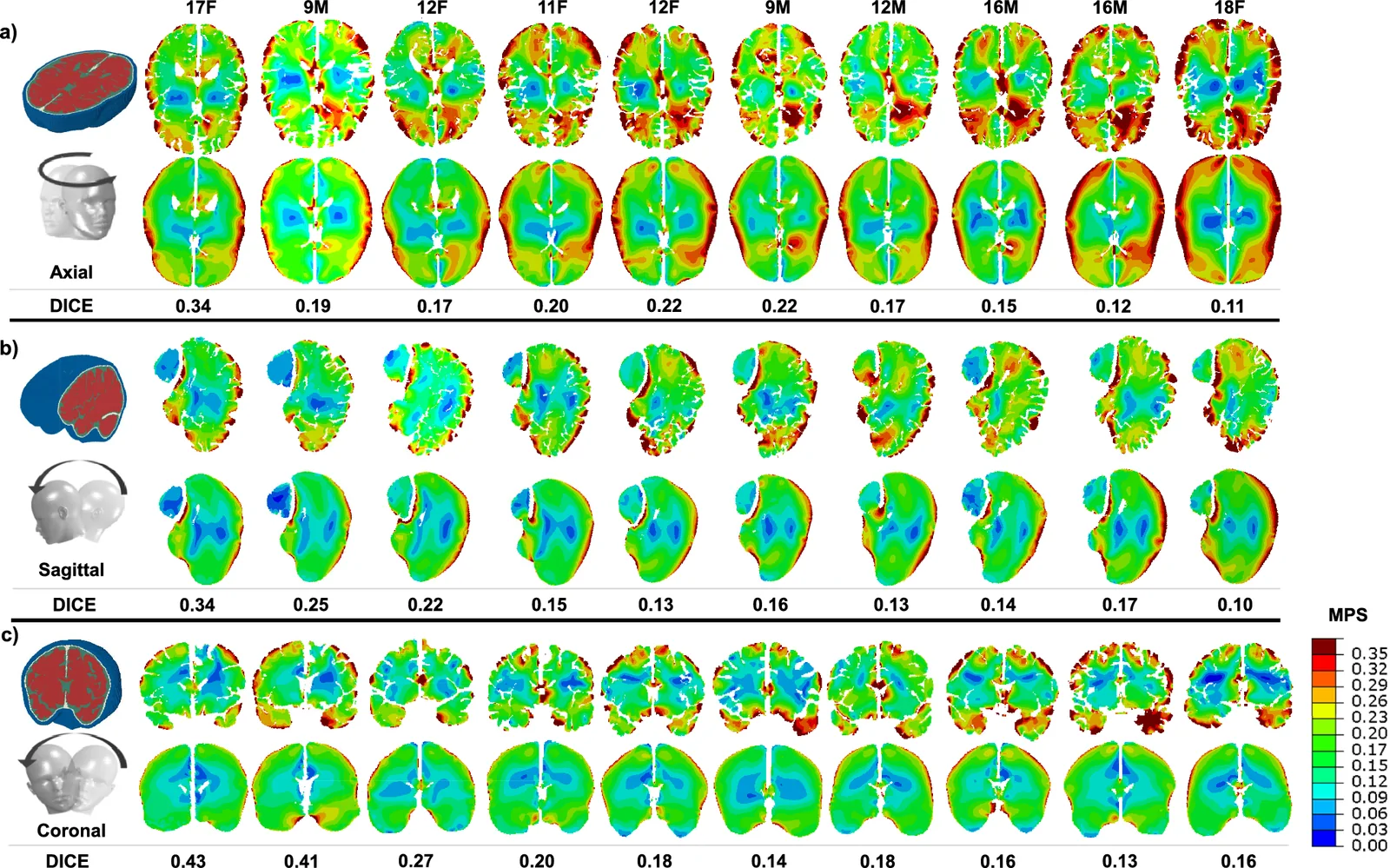
Peak maximum principal strain (MPS) contour plots shown for the lissencephalic and gyrencephalic models of multiple representative subjects under rotational acceleration about each principal axis: **a** axial, **b** sagittal, and **c** coronal

Both the gyrencephalic and lissencephalic models of all subjects experienced high strains on the outer cortical surface for all rotation directions. The high-strain regions tend to be located along the longer axis of the head, where the radius of curvature is high and the deformation of the brain is less constrained by the skull. The effect of the radius of curvature on the strain distribution is further discussed in Supplementary Section S5. The cortical surface regions with the highest strain in the subject-specific models include: i) the lateral surface for the axial rotation case (Figure 3a); ii) regions along the tentorium cerebelli, the convex curvature regions between the frontal and the temporal lobes, and the top cortical surface for sagittal rotations (Figure 3b); and iii) the lateral cortical surface and the inferior cortical surface for coronal rotations (Figure 3c). As discussed in Section 3.1.1, the skull-brain interface plays a significant role in governing the brain deformation, and the gyrencephalic model better captures the heterogeneity of the skull-brain interface. The variability in cortical folding between individuals also results in differences in subarachnoid CSF thickness. Since the high dissipation of the CSF has a cushioning effect on the cortical surface, the heterogeneous CSF thickness contributes to a higher heterogeneity in the brain tissue strain along the cortical surface in the gyrencephalic models. The effect of CSF thickness on brain strain is further discussed in Supplementary Section S4.

Strain concentrations are also found in the inner regions of the brain, which tend to be larger in magnitude in the gyrencephalic models compared to the lissencephalic models for all individuals and loading directions. These regions of higher strain tend to be located close to the ventricles and major sulci. For example, strains are concentrated between the anterior horns of the lateral ventricles and the lateral (Sylvian) sulcus for axial rotations of the gyrencephalic models and become increasingly larger with increasing head size or moment of inertia (MoI about the axial axis) (Wu et al. 2021b). Under sagittal rotations, the gyrencephalic models experience higher strains near the posterior horns of the lateral ventricles and the sulci in the frontal and temporal lobes. Similarly, under coronal rotations, the gyrencephalic models show high strain regions around the Sylvian sulcus in the frontal and temporal lobes, as well as in the corpus callosum between the tip of the falx and ventricles. The differences in the cortical fold geometry cause larger individual variations in the gyrencephalic models. These differences between individuals are not as pronounced in the lissencephalic models.

**Fig. 4:**
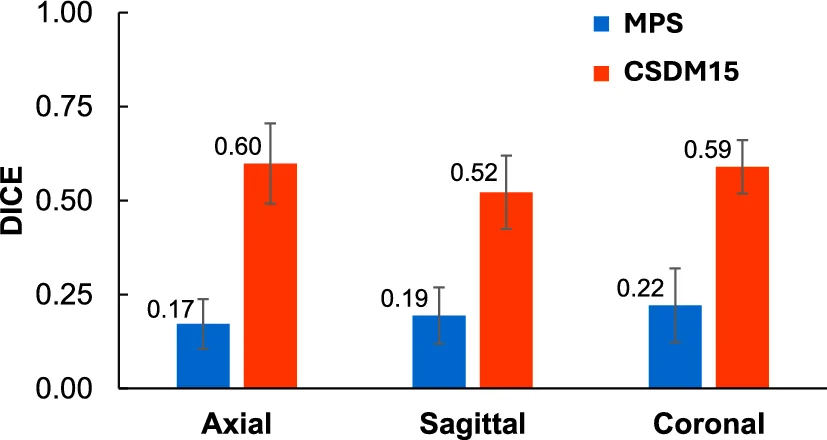
The similarity in the spatial distribution of the maximum principal strain (MPS) and cumulative strain damage measure (CSDM15) for the gyrencephalic and lissencephalic models is quantified using the Sørensen DICE coefficient (DICE) (with the error bars representing the standard deviation). We find no significant difference in the DICE coefficients for the different rotation directions

The DICE similarity coefficient (mean ± SD) between the gyrencephalic and lissencephalic models for the MPS distribution ranged between 0.17 ± 0.07 for axial rotations, 0.19 ± 0.08 for sagittal rotations, and 0.22 ± 0.10 for coronal rotations, underscoring the differences in the spatial strain distribution between the gyrencephalic and lissencephalic models (Figure 4). The CSDM15 DICE ranged between 0.60 ± 0.10, 0.52 ± 0.10, and 0.59 ± 0.08 for the axial, sagittal, and coronal rotations, respectively. The DICE for CSDM15 is higher than for MPS because CSDM15 uses a coarser discretization (steps of 1.0) to calculate the similarity as compared to the finer 0.025-step resolution used for MPS.

#### Effect of cortical folds on peak injury metrics

**Fig. 5:**
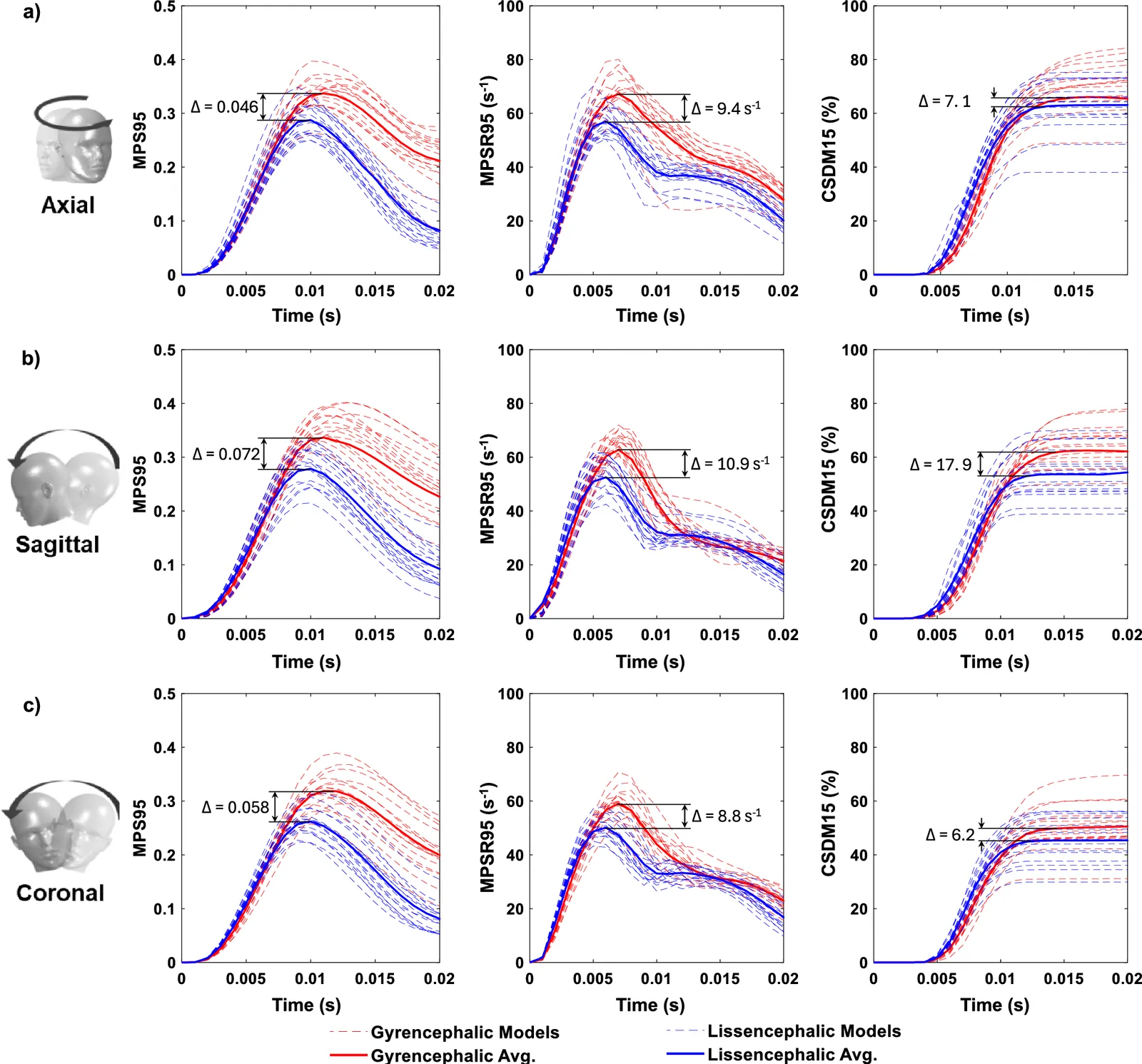
The MPS95, MPSR95, and CSDM15 time histories from the gyrencephalic (red) and lissencephalic (blue) models for all subjects are plotted for the three rotation directions: **a** axial, **b** sagittal, and **c** coronal. The individual responses are represented by the dashed curves, and the average responses are represented by the solid lines. The lissencephalic models underpredict the injury metrics for all subjects and for all loading directions

The time history plots of MPS95, MPSR95, and CSDM15 for all subjects and rotation directions show that the lissencephalic models consistently under-predicted all injury metrics (Figure 5). The time to peak for MPS95 and MPSR95 is also lower in the lissencephalic models for all loading directions (Figure 5), due to the differences in the location of the peak strains in the gyrencelphalic and lissenphalic models. The largest strain concentrations occur in the deep brain regions of the gyrencephalic models whereas the largest strains occur at the cortical surface in the lissencephalic models. This is also reflected in the CSDM15, which is higher in magnitude in the lissencephalic models at shorter times, before being surpassed by the gyrencephalic models at longer time points.

**Fig. 6:**
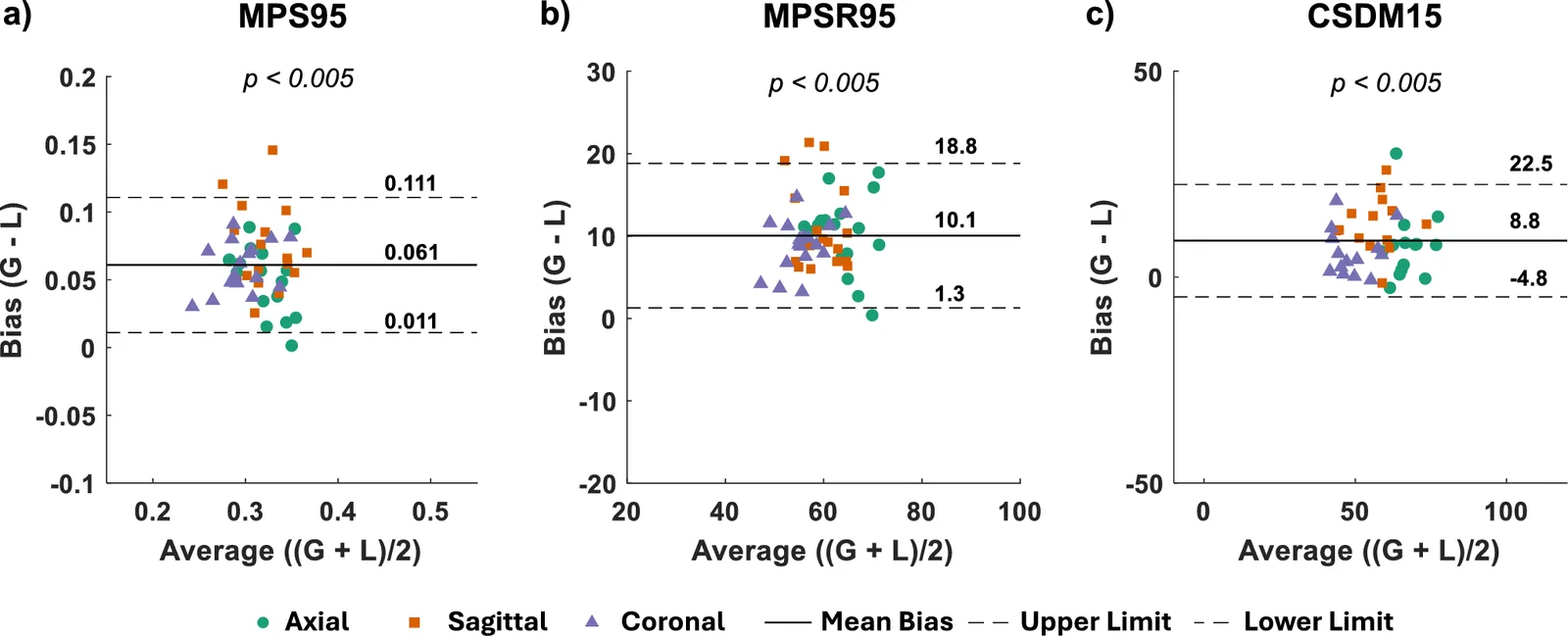
Bland-Altman analysis showing the mean bias and the limits of agreement (mean ± 1.96×SD) between the gyrencephalic (G) and lissencephalic (L) peak injury metrics: **a** MPS95, **b** MPSR95, and **c** CSDM15. The gyrencephalic models overpredict the injury metrics compared to the lissencephalic models with high statistical significance

The Bland-Altman analysis provides the bias (mean ± std. dev.) in the peak injury metric values between the gyrencephalic and lissenphalic models. The bias was found to be 0.061±0.026 (statistically significant, p<0.005) for MPS95, 10.05±4.48 s$^{-1}$ (p<0.005) for MPSR95, and 9.0±6.6 (p<0.005) for CSDM15 (Figure 6). Normalizing the bias by the maximum injury metric values experienced by the lissencephalic models provides the percentage bias (mean ± std. dev.) to be $21.7 \pm 9.1 \%$ for MPS95, $17.1\pm 7.6\%$ for MPSR95, and $14.4\pm 11.3\%$ for CSDM15, indicating that the inclusion of cortical folds leads to higher peak injury metric values. No proportional bias was observed in any injury metric.

### Effect of cortical folds on different regions of interest

In this section, we compare the gyrencephalic and lissencephalic simulation results for different regions of interest (ROI) to determine the regional extent of the influence of the cortical folds.

#### Cerebrum

The cerebrum is geometrically different between the gyrencephalic and the lissencephalic models. The region experienced overall higher MPS95, MPSR95, and CSDM15 in the gyrencephalic models than in the lissencephalic models for all three loading directions due to the strain concentrations near the cortical folds in the gyrencephalic models.

**Fig. 7:**
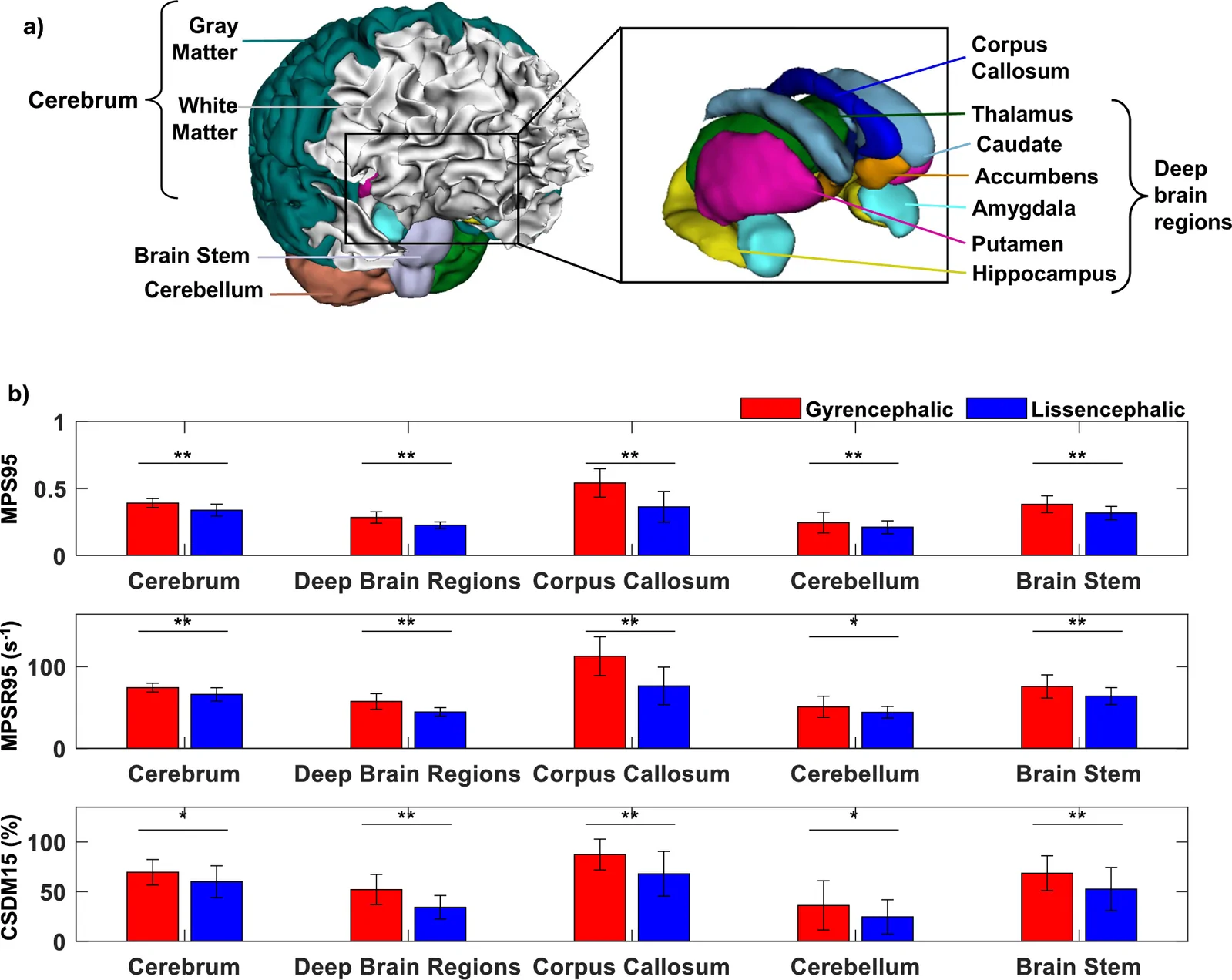
**a** The analyzed regions of interest (ROI) include the cerebrum, cerebellum, deep brain regions, corpus callosum, and brain stem. **b** The peak injury metrics (MPS95, MPSR95, and CSDM15) are shown for each ROI from the gyrencephalic and lissencephalic simulations (mean ± std. dev.). The statistical significance of the differences is shown (* indicates *p*<0.05, ** indicates *p*<0.005). These trends show that the gyrencephalic models predict higher peak injury metrics compared to the lissencephalic models with varied degrees of statistical significance

The MPS95, MPSR95, and CSDM15 (mean ± std. dev.) in the cerebrum were 0.39 ± 0.03, 74.6 ± 5.4 s$^{-1}$, and 69.5 ± 12.8 for the gyrencephalic models, respectively, and 0.34 ± 0.05, 66.9 ± 8.1 s$^{-1}$, and 60.0 ± 16.1 for the lissencephalic models, respectively (Figure 7b). From the Bland-Altman analysis, the bias (mean ± std. dev.) in the peak MPS95, peak MPSR95, and CSDM15 was found to be 0.053±0.043 (statistically significant, p<0.005), 8.53±8.56 s$^{-1}$ (p<0.005), and 6.6±6.4 (p<0.05), respectively (Supplementary Figure S12). The normalized bias values were $15.6 \pm 12.7\%$, $12.7 \pm 12.8\%$, and $18.2 \pm 17.5\%$ for the maximum MPS95, MPSR95, and CSDM15, respectively. No proportional bias was observed in any injury metric.

#### Deep brain regions

The deep brain regions, as defined in Section 2.4, are geometrically identical between the gyrencephalic and the lissencephalic models. However, the distance between the cortical surface and the deep brain regions differs between the models. This distance can be as small as ∼10 mm near the location of the deep sulci (e.g., Sylvian fissure, medial Cingulate sulci, etc.) in the gyrencephalic models, whereas it is at least ∼30 mm in the lissencephalic models. The deep brain regions experienced overall higher strain in the gyrencephalic models than in the lissencephalic models for all three loading directions (p<0.005) as a result of the strain concentrations at the base of the sulci.

The MPS95, MPSR95, and CSDM15 (mean ± std. dev.) in the deep brain regions were 0.28 ± 0.04, 57.4 ± 9.4 s$^{-1}$, and 52.2 ± 15.2 for the gyrencephalic models, respectively, and 0.23 ± 0.05, 44.6 ± 5.2 s$^{-1}$, and 32.3 ± 11.8 for the lissencephalic models, respectively (Figure 7b). We found the bias (mean ± std. dev.) in peak MPS95, peak MPSR95, and CSDM15 to be 0.057±0.038, 12.88±7.28 s$^{-1}$, and 17.1±12.1 (all p<0.005), respectively (Supplementary Figure S12). The normalized bias values were $25.3 \pm 17.0 \%$, $28.9 \pm 16.3 \%$ and $46.8 \pm 33.3 \%$ for the MPS95, MPSR95, and CSDM15, respectively. A proportional bias was observed for all injury metrics, with the bias increasing with the magnitude of the injury metric.

#### Corpus callosum

The body of the corpus callosum is geometrically the same between the gyrencephalic and the lissencephalic models. This region experiences some of the highest strains since it lies adjacent to the lateral ventricles and the falx cerebri, a stiff membrane extending from the skull. It also yields the highest biases between the two models for all loading directions due to its close proximity to medial sulci, such as the Cingulate sulcus. The strains were larger in the body of the corpus callosum in the gyrencephalic models than in the lissencephalic models for all three loading directions (p<0.005).

The MPS95, MPSR95, and CSDM15 (mean ± std. dev.) in the corpus callosum were 0.54 ± 0.10, 122.8 ± 23.7 s$^{-1}$, and 87.3 ± 15.6 for the gyrencephalic models, respectively, and 0.36 ± 0.11, 76.4 ± 22.9 s$^{-1}$, and 67.9 ± 22.6 for the lissencephalic models, respectively (Figure 7b). The bias (mean ± std. dev.) in peak MPS95, peak MPSR95, and CSDM15 was found to be 0.178±0.143, 38.05±32.65 s$^{-1}$, and 19.1±19.7 (all p<0.005), respectively (Supplementary Figure S12). The normalized bias values were $48.6 \pm 39.0 \%$, $49.4 \pm 42.4 \%$ and $52.3 \pm 53.8 \%$ for the MPS95, MPSR95, and CSDM15, respectively. We observe a proportional bias in MPS95 and MPSR95, with the bias increasing with magnitude in both injury metrics. The CSDM15 shows proportional bias under the axial and coronal loading directions, but not under the sagittal direction.

#### Cerebellum

The cerebellum is geometrically identical between the gyrencephalic and lissencephalic models. Given the spatial resolution of the models (1 mm), the cerebellar folds were not included in the models. This region experienced overall higher strain in the gyrencephalic models compared to the lissencephalic models for all three loading directions. Even though the cerebellum is separated from the cerebral cortical folds by the stiff tentorium cerebelli membrane, the cortical tissue in the inferior regions of the gyrencephalic models exhibited higher strain than in the lissencephalic models, which translated to greater deformation of the tentorium cerebelli and therefore, larger tissue strains in the cerebellum.

The MPS95, MPSR95, and CSDM15 (mean ± std. dev.) in the cerebellum were 0.25 ± 0.08, 50.8 ± 12.9 s$^{-1}$, and 36.1 ± 24.7 for the gyrencephalic models, respectively, and 0.21 ± 0.05, 44.2 ± 7.2 s$^{-1}$, and 24.6 ± 17.2 for the lissencephalic models, respectively (Figure 7b). The bias (mean ± std. dev.) in peak MPS95, peak MPSR95, and CSDM15 was found to be 0.035±0.042 (p<0.005), 7.18±6.51 s$^{-1}$ (p<0.05), and 10.8±11.5 (p<0.05), respectively (Supplementary Figure S12). The normalized bias values were $16.4 \pm 19.9 \%$, $16.2 \pm 14.7 \%$ and $29.6 \pm 31.6 \%$ for the MPS95, MPSR95, and CSDM15, respectively. We observe proportional bias in all injury metrics, with the bias increasing with the magnitude of the injury metric.

#### Brain stem

The brain stem is also geometrically identical between the gyrencephalic and the lissencephalic models and isolated from the cortical folds. This region experienced the second highest strain values compared to the other ROIs, with the corpus callosum being the highest. The strain in this region was higher in the gyrencephalic models than in the lissencephalic models for all three loading directions ($\textit{p}<0.005$). The largest strains occurred in the superior region of the brain stem (i.e., the midbrain), which is located close to the third ventricle. Since the strains are larger in the deep brain regions and near the ventricles in the gyrencephalic models, this also translated to larger strains in the superior region of the brain stem.

The MPS95, MPSR95, and CSDM15 (mean ± std. dev.) in the brain stem were 0.38 ± 0.06, 75.8 ± 14.1 s$^{-1}$, and 68.6 ± 17.5 for the gyrencephalic models, respectively, and 0.32 ± 0.05, 70.0 ± 10.4 s$^{-1}$, and 52.6 ± 21.8 for the lissencephalic models, respectively (Figure 7b). The bias (mean ± std. dev.) in peak MPS95, peak MPSR95, and CSDM15 was found to be 0.065±0.050, 11.17±10.85 $s^{-1}$, and 13.0±13.7 (all p<0.05), respectively (Supplementary Figure S12). The normalized bias values were $20.5 \pm 15.6 \%$, $17.4 \pm 16.9 \%$ and $35.5 \pm 37.5 \%$ for the MPS95, MPSR95, and CSDM15, respectively. We found no proportional bias in any injury metric.

These results show that the deformation of geometrically identical regions between the two models can be affected by the inclusion of cortical folds, namely the corpus callosum, brain stem, and deep brain regions. Regions further removed from the cortical folds, such as the cerebellum, were also found to experience larger strain in models that included cortical folds.

## Discussion

In this study, we aimed to determine the necessity of incorporating cortical folds into FE head models to accurately predict the risk of mTBI for rotational head acceleration events. We compared different mTBI injury metrics between gyrencephalic and lissencephalic FE models of 18 subjects aged 9–18 years. An idealized concussive loading profile with a rotational head acceleration of 10 krad/s$^2$ and rotational velocity of 60 rad/s about each principal anatomical axis was simulated with the models. In this section, we discuss our results with respect to other studies (Section 4.1), the limitations of our study (Section 4.2), and suggestions for future work (Section 4.2).

### Comparison with other studies

Several previous studies have investigated the role of cortical folds on mTBI injury metrics under head acceleration events and have found conflicting results. While some predicted higher injury metrics in gyrencephalic models (Mazurkiewicz et al. 2021; Fagan et al. 2020; Bakhtiarydavijani et al. 2021; He et al. 2022; Cloots et al. 2008), similar to the current study, others found higher strains in lissencephalic models (Ho and Kleiven 2009) or found different trends based on the metric (Song et al. 2015). In this section, we discuss the factors that may contribute to such discrepancies in the prediction of mTBI risk in these studies.

The studies vary in terms of the model length scale (full brain models or mesoscale models comprising of three to four cortical folds), applied loading condition, analyzed injury metrics, and the material properties. We first look at studies conducted at the whole-brain scale that found contrasting results to the current study. We only focus on those studies that subjected their head models to rotational kinematics (Ho and Kleiven 2009), which is shown to result in much higher brain strain as compared to translational kinematics (Zhang et al. 2006; Kleiven 2007). Several studies have only applied translational accelerations to their models (Fagan et al. 2020; Sáez et al. 2020; Mazurkiewicz et al. 2021); therefore, a direct comparison with this study is not possible. In most of these studies, the strains were quite low (less than 1–2 %) (Mazurkiewicz et al. 2021; Sáez et al. 2020).

One study that found contrasting results to the current study was conducted by Ho and Kleiven (Ho and Kleiven 2009). They subjected a 3D lissencephalic and gyrencephalic whole head model from a single subject to a rotational loading condition about the coronal and sagittal planes. For a 10 krad/s$^2$ (5 ms duration) applied angular acceleration, they found that most regions of the brain experienced higher strain in the lissencephalic model compared to the gyrencephalic model. Our study had a similar loading condition but found the opposite result. The differences in the results may arise from several factors. The Ho and Kleiven study reported the 100th percentile strain, whereas this study used the 95th percentile MPS. The chosen strain percentile has been shown to influence injury risk predictions (Fahlstedt et al. 2022). The modeling of the skull–brain interface also differed between the studies. The skull-brain interface in the Ho and Kleiven study included the dura mater, the subarachnoid CSF, and the Pia mater. The meninges were modeled with a non-linear elastic material model in the Ho and Kleiven study, which is different from the current study and may lead to differences in the response of the skull-brain interface. Furthermore, the subarachnoid CSF was modeled as an elastic fluid, and the Pia mater was modeled with shell elements with a shear stiffness of ∼50 kPa (Aimedieu and Grebe 2004), potentially making the skull-brain interface stiffer than the brain tissue. The Pia mater was not explicitly modeled in our study. A preliminary analysis on the effect of skull-brain interface stiffness on the brain strain in gyrencephalic and lissencephalic head models (Supplementary Section S3) shows that a stiffer subarachnoid space can lead to a prediction of lower strain in gyrencephalic models compared to lissencephalic models. This result is primarily due to the increased effective stiffness of the skull-brain interface in the gyrencephalic model, which leads to smaller brain deformations. There is currently no consensus on the best approach to use when modeling the skull–brain interface. While the choice of skull-brain interface in our model is based on previously validated models (Carlsen et al. 2021; Nakarmi et al. 2025; Fagan et al. 2020; Giudice et al. 2021; Mao et al. 2013), further work is needed to understand the role of the skull-brain interface stiffness on the brain strain. It is also worth mentioning that the quality of the segmentation and the ability to resolve the depth of sulci may also affect the results.

Another study that found higher brain strain in a lissencephalic model compared to a gyrencephalic model was conducted by Song et al. (Song et al. 2015). In this study, a cadaver head impact event by Nahum et al. (Nahum et al. 1977) was simulated with 2D gyrencephalic and lissencephalic head models. While the equivalent stress was found to be larger in the gyrencephalic model below the gyri and sulci, the maximum principal strain (100th percentile) was found to be lower in the gyrencephalic model in the analyzed brain regions, which included the cerebrum, corpus callosum, cerebellum, and the brain stem. The results of the study are shown for a total simulation time of 8 ms. This short time duration of the simulations may have contributed to the contrasting results with our study. In our study, the peak strains were larger in the lissencephalic models at short time durations but became larger in the gyrencephalic models at longer time durations. Therefore, if the simulations from the Song et al. study were extended over a longer period of time, the strains may have increased in the gyrencephalic model.

The finding from our study that the gyrencephalic models experience higher peak injury metrics is corroborated by most mesoscale studies (Bakhtiarydavijani et al. 2021; He et al. 2022; Cloots et al. 2008). There was one mesoscale modeling study by Saboori et el. (Saboori and Sadegh 2012) that found lower strains in a lissencephalic model compared to a gyrencephalic model. In the Saboori study, the subarachnoid CSF had a higher shear resistance (viscosity coefficient, η = 0.2) compared to our study (η = 0.001) and other meso-scale models (shear modulus = 0.12 Pa (Cloots et al. 2008)), which may have led to the conflicting results. An advantage of the mesoscale models is their ability to resolve cortical folds with a greater spatial resolution (∼0.1 mm in (He et al. 2022)) compared to macroscale models (∼1 mm), thereby better resolving the stresses at the base of the sulci. These studies generally find that as the depth of the sulci increases, the base of the sulci experiences higher strain concentrations (Bakhtiarydavijani et al. 2021; He et al. 2022; Cloots et al. 2008). However, since these models only capture the gyri and sulci at the cortical surface, they do not capture the effect of the cortical folds on the deformation of other brain regions, such as the deeper brain regions, which were also shown to be affected by the inclusion of cortical folds in the present study.

Comparing the results of our study with the existing literature, we find that the effect of modeling cortical folds is sensitive to other modeling parameters, especially the skull-brain interface. However, all studies conclude that modeling cortical folds significantly changes the spatial distribution and peak values of the strain-based injury metrics under head rotations. Studies that model the entire head, including the present study, show that regions distant from the cortical folds, such as the cerebellum or brain stem, also exhibit altered brain strains (Ho 2008; Mazurkiewicz et al. 2021; Song et al. 2015). Furthermore, studies on neurodegenerative conditions, such as Chronic Traumatic Encephalopathy (CTE), have found the accumulation of abnormal, hyperphosphorylated tau protein at the depth of the sulci (McKee et al. 2009). Tissue damage has also been found at the base of the sulci in patients who exhibited diffuse white matter degeneration following a closed head injury (Strich 1956). To capture the strain and stress concentrations that occur at the depth of the sulci, it is imperative to use head models that include cortical folds. However, additional studies are necessary to further assess the role of the skull-brain interface stiffness, the mode of loading, and other modeling parameters on the results.

### Limitations and future work

There are several limitations to our study. First, we only analyzed a single head acceleration magnitude of 10 krad/s$^2$, which lies towards the higher end of the range of measured impacts (Rowson et al. 2012; McIntosh et al. 2014). The loading magnitude was selected to be consistent with events with a high probability of concussion, but the differences in the wave dynamics of the gyrencephalic and lissencephalic models may be less pronounced for lower magnitude impacts. Also, the trend may be different for loading frequencies that coincide with the natural frequency of the lissencephalic models. Given the complex non-linear relationship between loading and peak injury metrics (Carlsen et al. 2021), future studies should assess the effect of modeling cortical folds over a wider range of loading conditions. The results of this study are also only applicable for the range of subjects studied. While the subjects encompassed a wide range of head sizes (cerebral volume: $1.1 \times 10^6- 1.4 \times 10^6$ mm$^3$) and shapes, it will be important to further assess the effect of these factors on the results.

The level of anatomical detail included in a computational model can also affect the results. The skull-brain interface in our models did not include the pia mater, which is a thin membrane covering the brain tissue, and the subarachnoid space was modeled as a soft solid rather than a fluid. Modeling the pia mater with nonlinear, experimentally based properties, as in Ho and Kleiven (2009), may increase the stiffness of the skull-brain interface. A stiffer skull-brain interface can result in reduced strains in gyrencephalic models compared to lissencephalic models (Supplementary Section S3). The most appropriate way to model the skull-brain interface is an active area of investigation, and some head models incorporate the pia mater (Ghajari et al. 2017; Ho and Kleiven 2009; Mao et al. 2013), whereas others do not (Giudice et al. 2021; Miller et al. 2016; Zhao et al. 2017). Prior studies have also reported mixed findings regarding the importance of including vasculature, with its effect on brain tissue deformation ranging from insignificant (Ho and Kleiven 2007) to considerable (Zhao and Ji 2020; Duckworth et al. 2022; Subramaniam et al. 2021). The FE head models used in this study did not include the brain vasculature. Although our modeling choices are consistent with some other FE modeling studies (Carlsen et al. 2021; Fagan et al. 2020; Upadhyay et al. 2022; Alshareef et al. 2021), the effect of these choices on the results should be further assessed in future studies.

The type of finite element mesh used in this study must also be considered when interpreting the results. We selected a uniform voxel mesh for our FE head models. While recent studies report small differences in peak strains between voxel and conformal mesh models under rotational head loading (Zhou et al. 2025), it will be important to further assess how the results of this study translate to conformal meshes, which have smoother material interfaces. Furthermore, the uniform voxel mesh does not allow for mesh adaptivity or higher mesh densities near high curvature regions, such as the cortical folds and ventricles, limiting the ability to capture the strain concentrations observed in meso-scale models (Cloots et al. 2008) or conformal meshes with higher mesh resolutions. The voxelized nature of the model and the tied interfaces at material boundaries also limited the amount of sliding that could occur between gyri, which may have further limited the strain localization. Future studies should assess the effect of these factors on the results.

Despite these limitations, the results of this study show that cortical folds have a significant effect on multiple injury metrics, across multiple subjects, and under different loading directions, highlighting the importance of incorporating cortical folds in future studies assessing mTBI risk.

## Supplementary Information

Below is the link to the electronic supplementary material.Supplementary file 1 (pdf 3126 KB)

## Data Availability

All data generated or analyzed during this study are included in the manuscript (and the supplementary files). The raw data will be made available upon reasonable request.
