# Supplementary material for: Effect of modeling subject-specific cortical folds on brain injury risk prediction under blunt impact loading
Source: Biomech Model Mechanobiol. 2026 Jun 19;25(4):70. doi: 10.1007/s10237-026-02095-1 (PMC13282220; doi:10.1007/s10237-026-02095-1)
Supplement: Supplementary file 1 — (pdf 3126 KB) [file 10237_2026_2095_MOESM1_ESM.pdf]

Supplementary Materials: Effect of modeling subject-specific cortical folds on brain injury risk prediction under blunt impact loading

Anu Tripathi[1], Alison Brooks[2], Traci Snedden[3], Peter Ferrazzano[4], Christian Franck[5], Rika Wright Carlsen[1*]

[1]Department of Engineering, Robert Morris University, Moon Township, PA, USA.
[2]Department of Orthopedics and Rehabilitation, University of Wisconsin–Madison, Madison, WI, USA.
[3]College of Nursing, University of Colorado Anschutz Medical Campus, Aurora, CO, USA.
[4]Waisman Center, University of Wisconsin–Madison, Madison, WI, USA.
[5]Department of Mechanical Engineering, University of Wisconsin–Madison, Madison, WI, USA.

*Corresponding author(s). E-mail(s): carlsen@rmu.edu;
Contributing authors: tripathia@rmu.edu; brooks@ortho.wisc.edu; traci.snedden@cuanschutz.edu; ferrazzano@pediatrics.wisc.edu; cfranck@wisc.edu;

S1 List of Acronyms

**CORA**	Correlation and Analysis
**CSDM**	Cumulative Strain Damage Measure
**CSF**	Cerebral Spinal Fluid
**DTI**	Diffusion Tensor Imaging
**FE**	Finite Element
**MRI**	Magnetic Resonance Imaging
**MPS**	Maximum Principal Strain
**MPSR**	Maximum Principal Strain Rate
**NDT**	Neutral Density Target
**NHTSA**	U.S. National Highway Traffic Safety Administration
**PLA**	Peak Linear Acceleration
**TBI**	Traumatic Brain Injury

S2 Validation of the finite element head model

The brain deformation response of the FE head models was evaluated against the following experimental datasets: (a) cadaveric head impact tests by Hardy et al. (Hardy et al, 2007) and (b) *in vivo* strain measurements from tagged MRI of mild head accelerations (Knutsen et al, 2020).

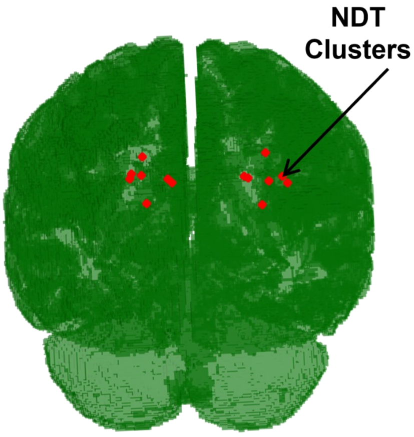

Fig. S1: Representative NDT clusters location assignments of specimen C288 in the finite element model based on reported coordinates from Hardy et al. 2007 (Hardy et al, 2007).

S2.1 Validation against Hardy cadaveric head impact study

In this study, four tests from two subjects (Table S1) were selected from the cadaveric head impact tests of Hardy et al. (Hardy et al, 2007) to evaluate the brain deformation response from a representative FE head model (from an 18 year old male). In these tests, cadaveric heads were impacted at either the occipital region, the left-side temporal region, or the left-side parietal region. The skull-brain relative motion was captured by high-speed X-ray with two implanted clusters of neutral density targets (NDTs). The average tissue strains and NDT displacements from the experiments were reanalyzed by Zhou et al. (Zhou et al, 2018, 2020), and we used this reanalyzed data to evaluate our simulations (instead of the original data from Hardy et al.). In our simulations, the finite element model was scaled uniformly in each direction to match the head size of the experimental subjects, and the location of the NDTs was defined based on the reported initial locations (Zhou et al, 2018) shown in Figure S1. The NDTs in each cluster were adjusted slightly such that all the NDTs were located in the brain tissue and the relative distance between the NDTS remained unchanged.

Table S1: Selected cadaveric experimental tests and subject dimensions from Hardy et al. 2007 (Hardy et al, 2007) used to evaluate the FE model.

Specimen and Test		Impact	Plane	Region	Helmet	Head dimensions	
						Depth	Breadth
C288	T3	offset	median	occipital	n	18.4 cm	13.3 cm
C380	T2	offset	horizontal	parietal lt.	y	18.5 cm	16.0 cm
	T4	offset			n		
	T6	aligned	coronal	temporal lt.	n		

The time history of average maximum principal strain (Green-Lagrange) at the NDT clusters and the NDT displacements were exported from the simulations and compared with the experimental data. CORA (Correlation and Analysis) scores that quantitatively compare the time history from the simulation results and the experimental data were evaluated using standard CORA parameters (Giordano and Kleiven, 2016). The results were plotted against results from other finite element models (Zhou et al, 2018; Menghani et al, 2023; Nakarmi et al, 2025) as shown in Figures S2 - S6. The strain response of our FE model showed good agreement and high correlations with the experimental data, with CORA scores ranging from 0.693 (fair) to 0.910 (excellent), as shown in Figure S2. The CORA scores of the NDT displacements ranged from 0.40 (marginal) to 0.860 (excellent). The average CORA score of the NDT displacement falls within the fair and good categories of the biofidelity scale, as shown in Table S2.

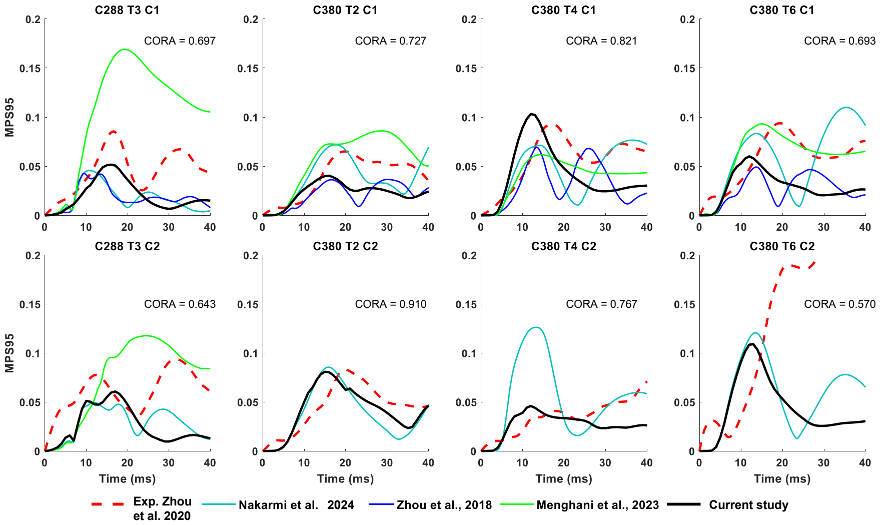

Fig. S2: Comparison of the maximum principal strain of the NDT clusters between our simulated results and experimental results from Hardy et al. (Zhou et al, 2020) and other finite element modeling studies (Menghani et al, 2023; Zhou et al, 2018; Nakarmi et al, 2025).

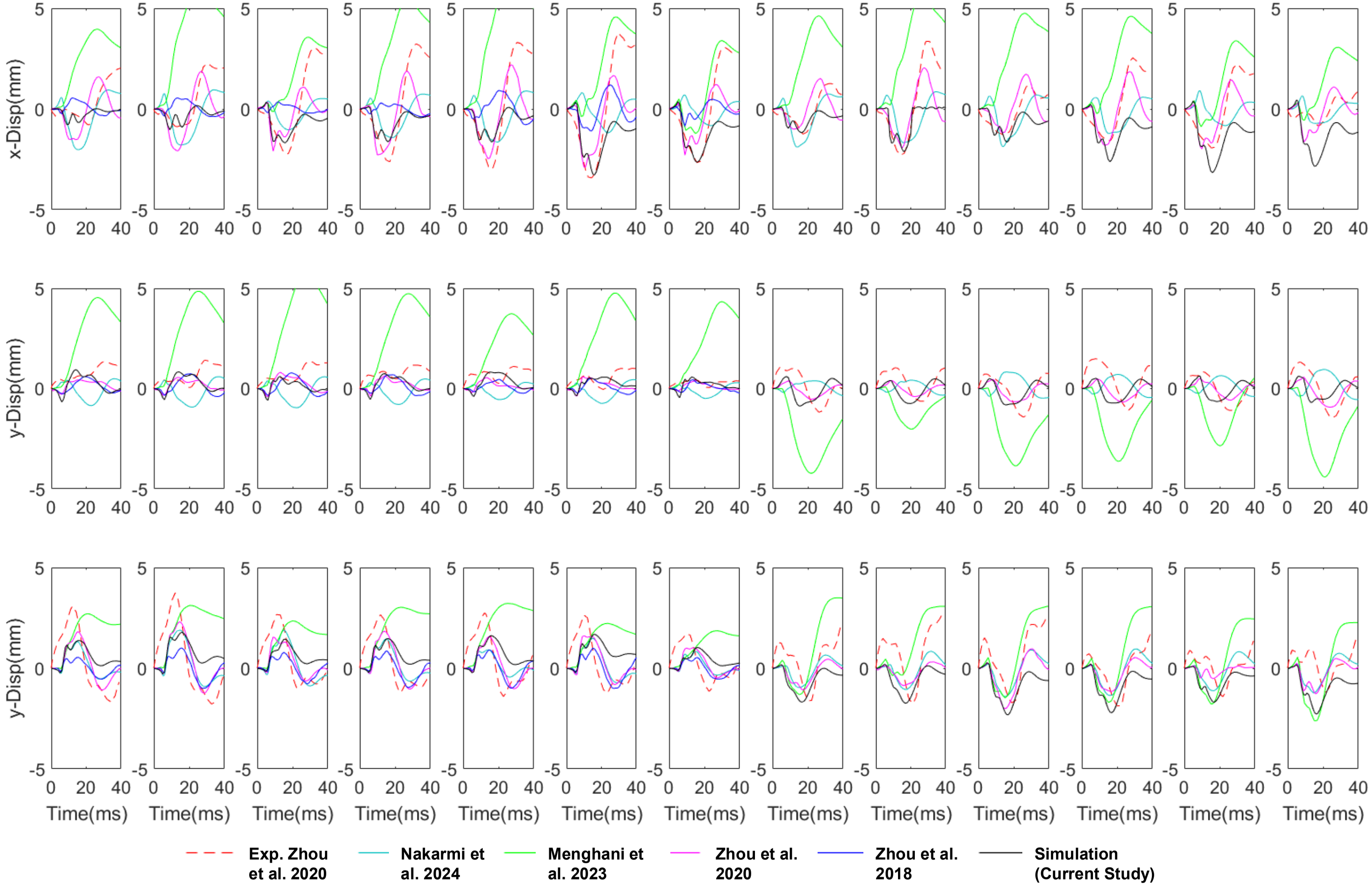

Fig. S3: Comparison of NDT displacements between our simulated results and experimental results from Hardy (Zhou et al, 2020) and other FEA studies (Zhou et al, 2020; Menghani et al, 2023; Zhou et al, 2018; Nakarmi et al, 2025) for test case C288-T3.

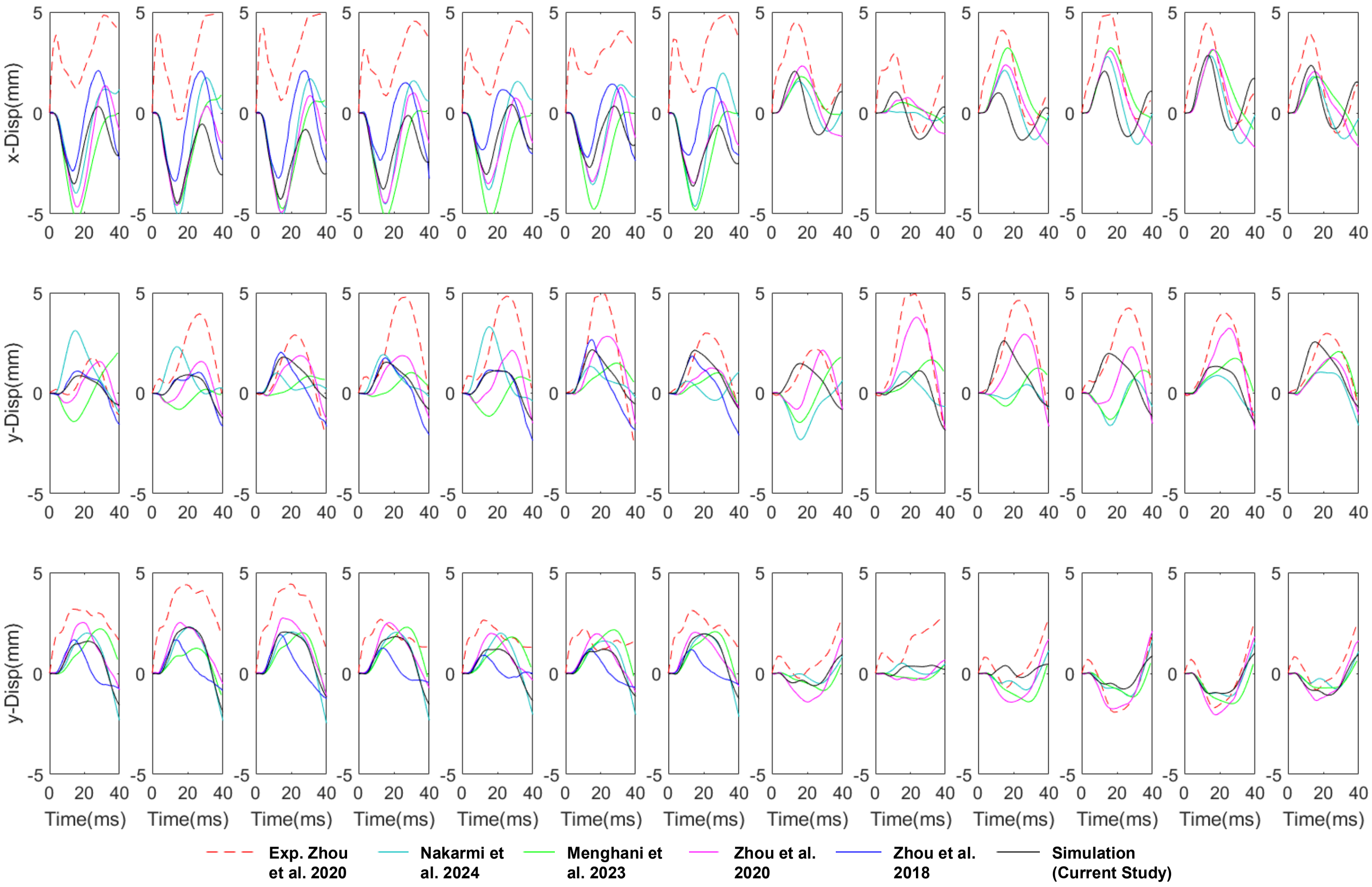

Fig. S4: Comparison of NDT displacements between our simulated results and experimental results from Hardy (Zhou et al, 2020) and other FEA studies (Zhou et al, 2020; Menghani et al, 2023; Zhou et al, 2018; Nakarmi et al, 2025) for test case C380-T2.

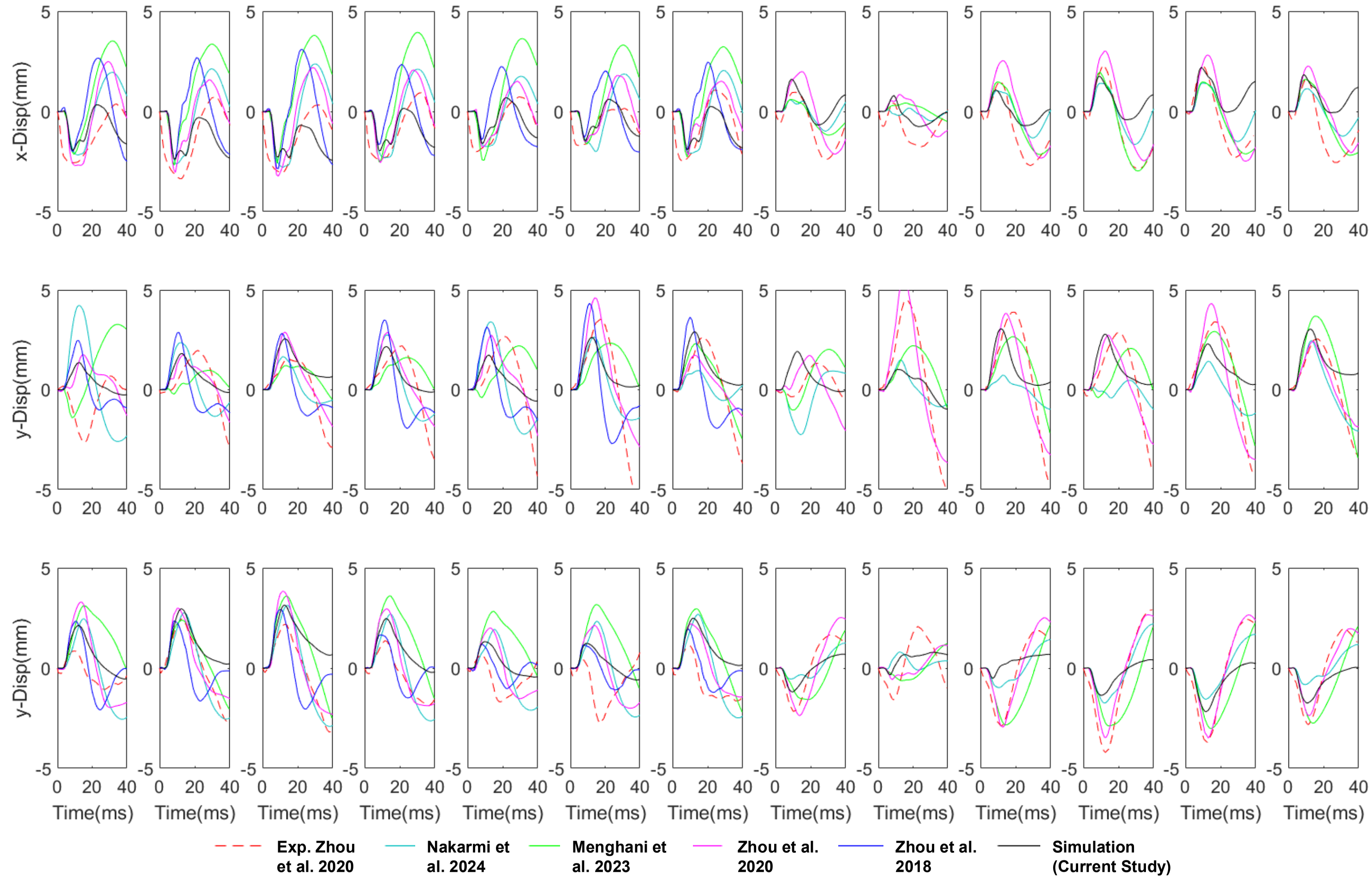

Fig. S5: Comparison of NDT displacements between our simulated results and experimental results from Hardy (Zhou et al, 2020) and other FEA studies (Zhou et al, 2020; Menghani et al, 2023; Zhou et al, 2018; Nakarmi et al, 2025) for test case C380-T4.

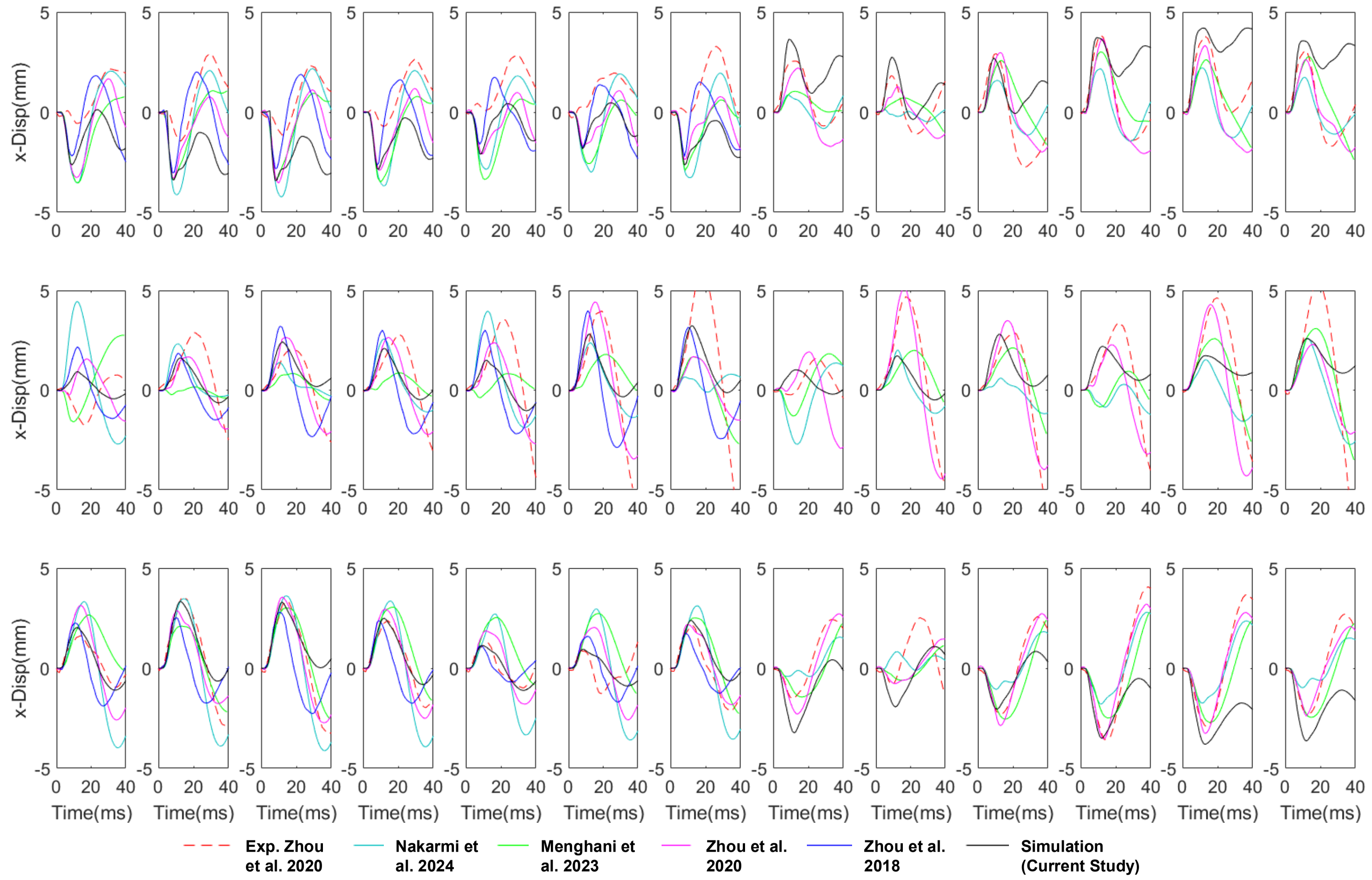

Fig. S6: Comparison of NDT displacements between our simulated results and experimental results from Hardy (Zhou et al, 2020) and other FEA studies (Zhou et al, 2020; Menghani et al, 2023; Zhou et al, 2018; Nakarmi et al, 2025) for test case C380-T6.

Table S2: CORA scores of NDT displacements compared with experimental results.

		C288T3	C380T2	C380T4	C380T6
X-displacement	NDT1	0.747	0.487	0.771	0.669
	NDT2	0.77555	0.4835	0.77173	0.67966
	NDT3	0.74517	0.59206	0.77055	0.74118
	NDT4	0.58057	0.55822	0.83981	0.66229
	NDT5	0.61107	0.55145	0.84328	0.68553
	NDT6	0.61621	0.50858	0.83083	0.77571
	NDT7	0.50787	0.47078	0.63699	0.5962
	NDT8	0.49786	0.51413	0.8595	0.76486
	NDT9	0.83306	0.61351	0.65787	0.61511
	NDT10	0.61881	0.39951	0.57162	0.40227
	NDT11	0.84587	0.6843	0.6832	0.69564
	NDT12	0.58257	0.68988	0.73912	0.7488
	NDT13	0.52516	0.74059	0.77762	0.65912
	NDT14	0.79135	0.67091	0.68902	0.76004
Y-displacement	NDT1	0.456	0.59	0.423	0.377
	NDT2	0.77555	0.4835	0.77173	0.67966
	NDT3	0.74517	0.59206	0.77055	0.74118
	NDT4	0.58057	0.55822	0.83981	0.66229
	NDT5	0.61107	0.55145	0.84328	0.68553
	NDT6	0.61621	0.50858	0.83083	0.77571
	NDT7	0.50787	0.47078	0.63699	0.5962
	NDT8	0.49786	0.51413	0.8595	0.76486
	NDT9	0.83306	0.61351	0.65787	0.61511
	NDT10	0.61881	0.39951	0.57162	0.40227
	NDT11	0.84587	0.6843	0.6832	0.69564
	NDT12	0.58257	0.68988	0.73912	0.7488
	NDT13	0.52516	0.74059	0.77762	0.65912
	NDT14	0.79135	0.67091	0.68902	0.76004
Z-displacement	NDT1	0.638	0.637	0.551	0.652
	NDT2	0.77555	0.4835	0.77173	0.67966
	NDT3	0.74517	0.59206	0.77055	0.74118
	NDT4	0.58057	0.55822	0.83981	0.66229
	NDT5	0.61107	0.55145	0.84328	0.68553
	NDT6	0.61621	0.50858	0.83083	0.77571
	NDT7	0.50787	0.47078	0.63699	0.5962
	NDT8	0.49786	0.51413	0.8595	0.76486
	NDT9	0.83306	0.61351	0.65787	0.61511
	NDT10	0.61881	0.39951	0.57162	0.40227
	NDT11	0.84587	0.6843	0.6832	0.69564
	NDT12	0.58257	0.68988	0.73912	0.7488
	NDT13	0.52516	0.74059	0.77762	0.65912
	NDT14	0.79135	0.67091	0.68902	0.76004
Average		0.6562	0.5752	0.7439	0.6759

S2.2 Validation against in vivo experiments using tagged MRI

The strain response from finite element models of two representative subjects (9 yr. old male and an 18 yr. old male) were compared to the strains measured from an *in vivo* tagged magnetic resonance imaging (MRI) experiment of a mild, non-injurious head impact (Knutsen et al, 2020). The subjects were selected to represent the range of head shapes and sizes in our cohort. The angular velocity of the head and the direction of neck rotation are shown in Figure S7. A qualitative comparison between the MPS contours of the tagged MRI experiment and simulation is shown in Figure S8a, along with a comparison to other FE head models validated using the same experimental test (Nakarmi et al, 2025; Upadhyay et al, 2022; Alshareef et al, 2021). The quantitative comparison between the maximum principal strain (MPS) from the experiment and our simulation is shown in Figure S8b. The CORA score between the simulations and experimental MPS95 is found to be 0.674, which lies in the 'fair' range. For the very low strain and strain rates used in the experiment, the simulation under-predicts the principal strains. The peak MPS95 for the experiment and simulation

are 0.038 and 0.019, respectively. The peak MPS95 occurred at the same time instance (45 ms) in both the simulation and experiment.

Our models under-predict the strains as compared to the experimental data. A conformal mesh model from Nakarmi et al. (Nakarmi et al, 2025), which uses the same material properties as our models, also under-predicts the experimental strain, indicating that the material response is overly stiff for this lower strain regime. Our models were slightly stiffer than the conformal mesh model, which could arise from the jagged interfaces in the voxel mesh. On the other hand, Alshareef et al. (Alshareef et al, 2021) also used a voxel mesh, but they incorporated brain material properties directly from magnetic resonance elastography data and over-predicted the brain strain. Upadhyay et al. (Upadhyay et al, 2022) incorporated non-linear viscoelasticity and simulated the experiment using the material point method, resulting in very good agreement with the experimental data.

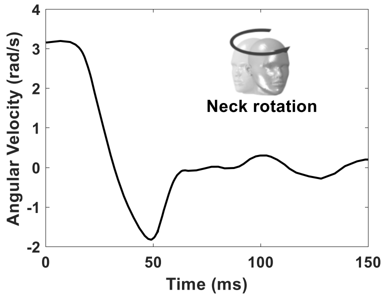

Fig. S7: Angular velocity of the head for the neck rotation case.

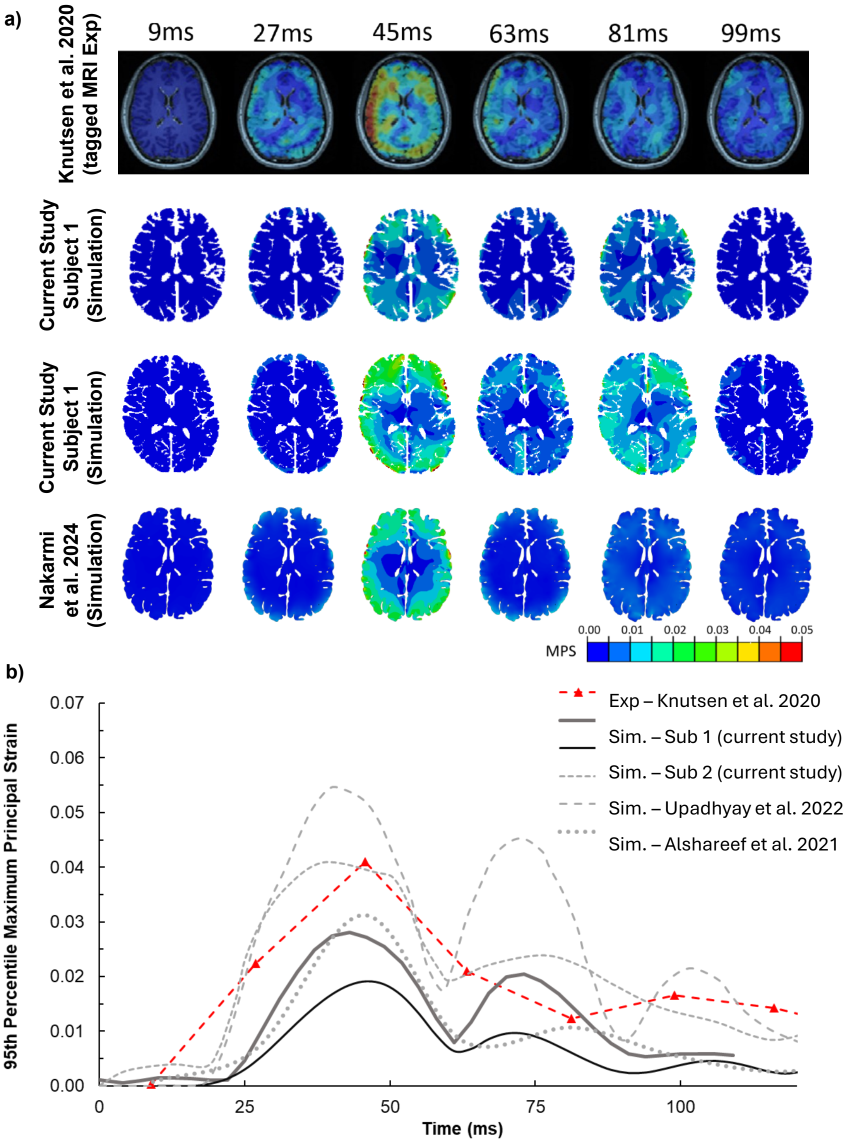

Fig. S8: a) Comparison of the evolution of MPS in the brain from the in vivo experiment (Knutsen et al, 2020) (top row) and our simulation (bottom two rows); b) MPS95 from the experiment (Knutsen et al, 2020) (red) and simulations of two subject models (black, gray solid) in our study showing the upper and lower bounds, from a conformal mesh model with the same material (Nakarmi et al, 2025) (gray dashed), and other recent head models with different brain material properties (Alshareef et al, 2021; Upadhyay et al, 2022).

S3 Effect of subarachnoid space stiffness

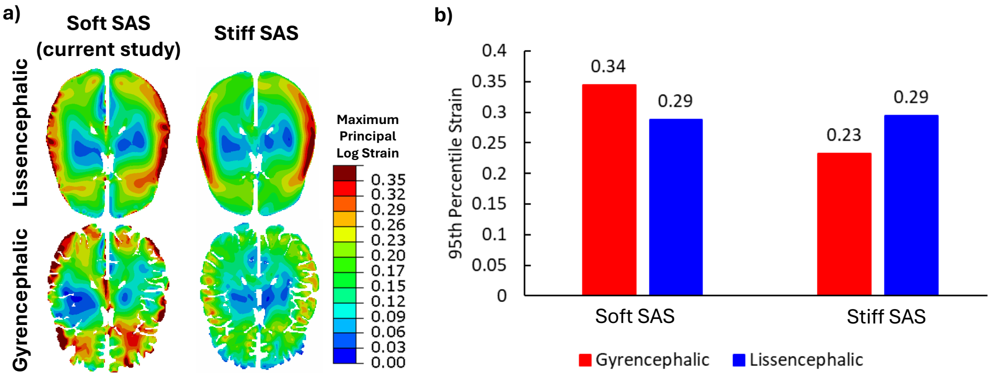

Fig. S9: a) Maximum principal strain distribution showing the effect of subarachnoid space (SAS) stiffness. b) The model with stiffer SAS has lower strains in the gyrencephalic model, whereas the lissencephalic model experiences minimal effect. The effect of a stiffer skull-brain interface was assessed by increasing the stiffness of the sub-arachnoid space (SAS) CSF to 20 kPa (Neo-Hookean model), as seen in other studies (Ghajari et al, 2017). This results in a stiffer skull-brain interface compared to the long-term modulus of the brain, which was 6400 Pa (Carlsen et al, 2021). Since the sulci in the gyrencephalic models are filled with CSF, the increase in the effective stiffness reduces the overall strain in the gyrencephalic model more significantly as compared to the lissencephalic model, which experiences a smaller decrease in the strain. This results in higher strains in the lissencephalic model compared to the gyrencephalic model for the stiff SAS.

S4 Effect of CSF layer thickness on brain tissue strain

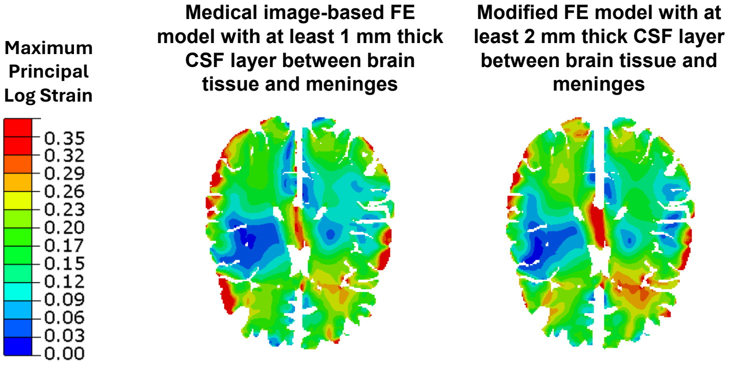

Fig. S10: Maximum principal strain distribution showing the effect of CSF layer thickness. The model with a thicker CSF exhibits higher strains in deep brain regions and lower strains on the cortical surface as compared to the model with a thinner CSF layer.

S5 Effect of radius of curvature on high strain regions

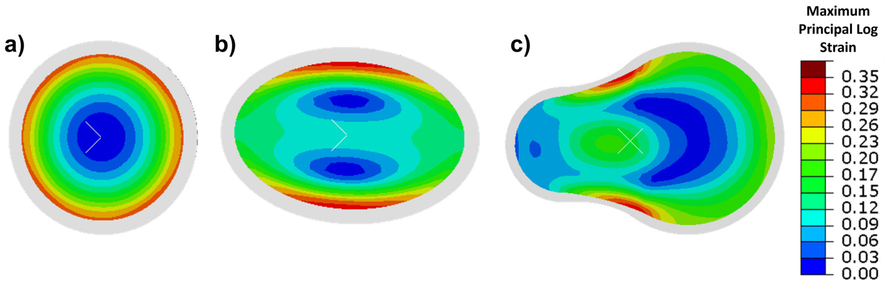

Fig. S11: Maximum principal strain distribution showing the effect of radius of curvature through idealized shapes: a) a sphere under axial rotation of the skull (gray) shows a uniform high strain region on the outer surface, b) an ellipsoid under axial rotation of the skull (gray) shows high strain regions perpendicular to the small axis of the ellipse where the radius of curvature is high, allowing the brain tissue to deform more; and c) A 3D body with variable curvature shows high strain regions where the radius of curvature is high or convex, allowing the brain tissue to deform more.

S6 Effect of cortical folds on ROI strains

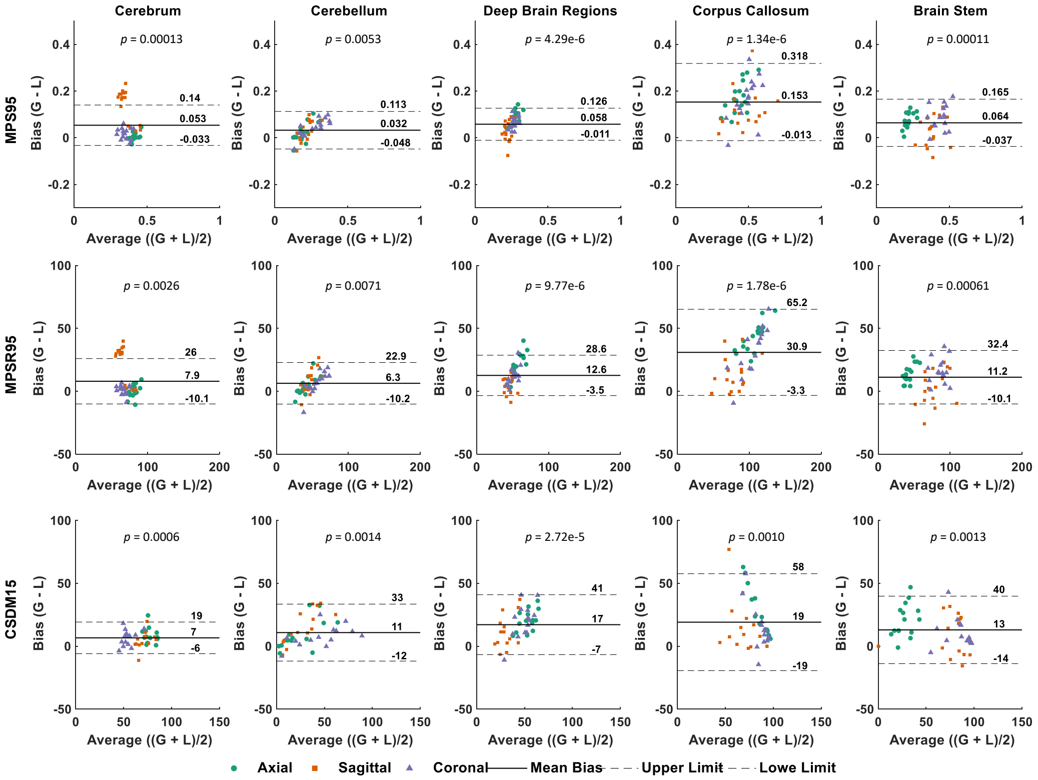

Fig. S12: Bland-Altman analysis showing the mean bias and the limits of agreement (mean ± 1.96×SD) between the gyrencephalic (G) and lissencephalic (L) peak injury metrics: MPS95, MPSR95, and CSDM15. A statistically significant overprediction of injury metrics was observed in the gyrencephalic models relative to the lissencephalic models.

References

Alshareef A, Knutsen AK, Johnson CL, et al (2021) Integrating material properties from magnetic resonance elastography into subject-specific computational models for the human brain. Brain multiphysics 2:100038

Carlsen RW, Fawzi AL, Wan Y, et al (2021) A quantitative relationship between rotational head kinematics and brain tissue strain from a 2-d parametric finite element analysis. Brain Multiphysics 2:100024

Ghajari M, Hellyer PJ, Sharp DJ (2017) Computational modelling of traumatic brain injury predicts the location of chronic traumatic encephalopathy pathology. Brain 140(2):333–343

Giordano C, Kleiven S (2016) Development of an unbiased validation protocol to assess the biofidelity of finite element head models used in prediction of traumatic brain injury. Tech. rep., SAE Technical Paper

Hardy WN, Mason MJ, Foster CD, et al (2007) A study of the response of the human cadaver head to impact. Stapp Car Crash Journal 51:17

Knutsen AK, Gomez AD, Gangolli M, et al (2020) In vivo estimates of axonal stretch and 3D brain deformation during mild head impact. Brain Multiphysics 1:100015

Menghani RR, Das A, Kraft RH (2023) A sensor-enabled cloud-based computing platform for computational brain biomechanics. Computer Methods and Programs in Biomedicine 233:107470

Nakarmi S, Wang Y, Fawzi AL, et al (2025) Estimating brain injury risk from shipborne underwater blasts using a high-fidelity finite element head model. Military medicine 190(1-2):e202–e210

Upadhyay K, Alshareef A, Knutsen AK, et al (2022) Development and validation of subject-specific 3d human head models based on a nonlinear visco-hyperelastic constitutive framework. Journal of the Royal Society Interface 19(195):20220561

Zhou Z, Li X, Kleiven S, et al (2018) A reanalysis of experimental brain strain data: Implication for finite element head model validation. SAE Technical Paper, 2018-22-0007

Zhou Z, Li X, Kleiven S, et al (2020) Brain strain from motion of sparse markers. Tech. rep., SAE Technical Paper
